# Multilayered Forensic Protocol Based on In-Depth Mass Spectrometry Techniques for the Investigation of Suspicious Drums of Oils with the 2019–2022 Brazilian Oil Spill Disaster

**DOI:** 10.1021/acsomega.5c07056

**Published:** 2025-10-01

**Authors:** Jhonattas Carvalho Carregosa, Mirele Santana Sá, Jandyson Machado Santos, Alberto Wisniewski

**Affiliations:** † Petroleum and Energy from Biomass Research Group (PEB), Department of Chemistry, Federal University of Sergipe, Jardim Rosa Elze, São Cristóvão, Sergipe 49107-230, Brazil; ‡ Department of Chemistry, Rural Federal University of Pernambuco, Dois irmãos, Recife, Sergipe 52171-900, Brazil

## Abstract

In 2019, the northeast coast of Brazil experienced the country’s largest environmental disaster involving a mysterious oil spill. At the same time, two drums containing an oily substance were found ashore on the coasts of Sergipe and Rio Grande do Norte. Since oil spills are often unavoidable, it was necessary to assess the possibility of simultaneous spills. In this context, a new multilayered protocol is proposed in this work to determine the similarity or dissimilarity between the oily substance in the drums and the suspected samples. Due to geographic proximity, samples from the Sergipe–Alagoas basin were also included as suspects, alongside samples collected from the oil spill. Data obtained from reconstructed-ion chromatograms using *m*/*z* 85 and *m*/*z* 192, as well as hopane and sterane biomarkers analyzed by gas chromatography-based techniques, trace elements determined by energy-dispersive X-ray analysis (EDX), and ultrahigh-resolution mass spectrometry (UHR MS) data, confirmed that the spilled oils originated from the same event. Furthermore, UHR MS, as an additional analytical layer, helped definitively establish the similarity or dissimilarity relationships between the sample sets when analyzed through principal component analysis (PCA) and hierarchical clustering analysis (HCA). These results suggest that the oil from the drums does not share similarities with oils produced in the Sergipe–Alagoas basin. However, it does show similarities to mysterious oils found on Brazilian northeast beaches in 2019.

## Graphical abstract

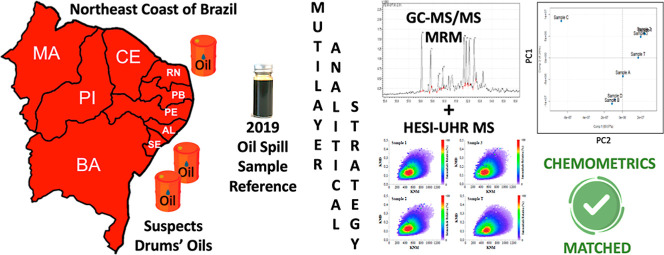

## 1 Introduction

Oil spills present a significant global challenge, and identification of their sources is of paramount importance. In forensic investigations, a combination of specific and nonspecific analyses is employed to characterize oil samples, which helps determine the nature of the product and suggest its probable origin.^1^ Among the analytical techniques used to characterize petroleum and its derivatives, the specific identification of biomarkers is particularly valuable for establishing the chemical fingerprints of the oils under investigation. Biomarkers found in oils, rocks, and sediments are chemically stable and undergo little to no alteration from their biogenic precursors.^2^

Analysis of compounds classified as biomarkers provides crucial information regarding the contribution of organic matter, the geological era of formation, the state of thermal evolution, the paleo-deposition environment, and the current degree of biodegradation.^3^ For this purpose, techniques based on gas chromatography (GC) with or without hyphenation and mass spectrometry (MS) have been the most widely used and recommended. Examples include gas chromatography–mass spectrometry (GC–MS) and gas chromatography with flame ionization detection (GC-FID), as stipulated in the CEN methodology.^4,5^ Theoretically, each petroleum formed over geological eras possesses a unique composition that can be differentiated by specific techniques such as those mentioned above. Nevertheless, real-world spills present additional analytical challenges due to factors like oil mixtures (blending)^6^ or processed oil that has undergone viscosity reduction.^1^ In both scenarios, significant alterations are observed that can impact forensic analysis. Because this data is vital for determining the source and geochemical origin of the spill, having reliable methods for unequivocal characterization, identification, and source attribution is essential for withstanding legal scrutiny.^7–9^

The single quadrupole mass analyzer (QMS) has certain limitations in sensitivity and selectivity. For example, when assessing the performance of multiple reaction monitoring (MRM), selected ion monitoring (SIM), and SCAN modes for quantifying polycyclic aromatic hydrocarbons (PAHs) in oil samples, the MRM mode is considered the most suitable for this purpose.^10^ In gas chromatography–tandem mass spectrometry (GC–MS/MS), MRM reduces issues with interfering chromatographic peaks and enhances the signal-to-noise (S/N) ratio compared to the SIM mode. This is especially important when analyzing complex natural mixtures like crude oils in forensic oil spill investigations.^11,12^ Because of these benefits, GC–MS/MS has become more popular for this application, as it offers more selective and sensitive methods for identifying oil biomarkers such as PAHs, terpanes, and steranes.^13,14^ However, crude oil is an extremely complex mixture containing tens of thousands of hydrocarbons and nonhydrocarbon compounds. These range from small, simple, volatile molecules like methane to very large, complex, nonvolatile, colloidally dispersed macromolecules, such as asphaltenes.^15^ In this setting, gas chromatography-based methods struggle to fully characterize polar and high-molecular-mass compounds. As a result, more advanced mass analyzers, like ultrahigh-resolution mass spectrometry (UHR MS), have become increasingly important in forensic oil chemistry.^16,17^

Forensic investigations were initiated following the oil spill incident in the northeastern region of Brazil, which began on August 30, 2019. From the first appearance of oil slicks on the beaches until March 19, 2020, approximately 1009 locations (with a minimum distance of 1 km between them) were officially registered as being affected by the oil.^13^ This incident, which represents the most significant environmental disaster caused by an oil spill in the country’s history, inflicted extensive damage to ecosystems and had significant socioeconomic impacts on the affected areas.^18^ Environmental studies and reports on the consequences for marine biodiversity and local populations highlighted the full extent of the damage, as well as the lack of definitive information regarding the origin of the material.^19,20^

Lourenço et al. (2020) conducted a study on the 2019 oil spill that impacted the Brazilian coast. Using GC-FID and GC-QMS (operating in SIM and SCAN modes), they analyzed 11 oil slick samples collected from various beaches in September 2019. The results, based on biomarker identification and their corresponding ratios, indicated a similarity pattern among 10 of the 11 samples.^21^ According to a recent publication by Zacharias, Gama, and Fornaro (2021), the investigations and published studies have proposed two main hypotheses for the origin of the oil from the Brazilian coast spill: (a) an unidentified vessel spilling oil into the ocean approximately 700 km from the coast, or (b) a slow oil leak from an old or new shipwreck.^22^

Due to the proximity of the Sergipe–Alagoas (SE–AL) basin to the coasts of Bahia, Alagoas, Pernambuco, and Sergipe (described as the states most affected in terms of oil volume),^20^ a hypothesis that oils from this basin could be related to the disaster arose. However, in 2021, our group used a GC–MS/MS methodology to demonstrate no correlation with oils produced in the SE–AL basin. Furthermore, our findings, corroborated by other researchers, identified characteristics consistent with oils produced in Venezuelan oil fields.^13,23^

Conventional forensic oil spill analysis, often relying on GC-FID and GC-QMS, is highly effective for characterizing petroleum samples. However, these techniques have limitations when dealing with highly degraded oils, complex mixtures, or samples with low concentrations of diagnostic compounds. This is because GC-FID provides a general hydrocarbon profile but lacks the specificity to resolve coeluting compounds. Similarly, GC-QMS in SIM mode, while more selective, can still be prone to interference from background signals, especially in environmentally weathered samples. The presence of multiple, nonrelated spills or a spill from a processed oil source can further complicate the interpretation of data, potentially leading to an inaccurate conclusion of “no correlation”.

Knowing that oil spills are common and often inevitable in industrial processes, it is necessary to consider the possible occurrence of simultaneous spills during the same period, starting on August 30, 2019. In this context, a drum was found in the exact location and period of the oil spill in the state of Sergipe. However, after analyzing the biomarkers using conventional methodology (GC-FID and GC-QMS), local authorities concluded that the oil content had “no correlation” with the incident. This scenario raises the following questions: What if this specific case is one where conventional methodology is not sufficient? Would data from a more advanced technique, such as GC–MS/MS-MRM, lead to the same conclusion? And would the polar compounds also corroborate this result?

Therefore, the goal of this study was to develop an advanced multilayered protocol (with three levels) for analyzing both nonpolar and polar biomarkers in samples associated with the oil spill in Sergipe, Brazil, during 2019–2020. The layers include (1) characterization of organic nonpolar biomarkers using reconstructed ion chromatograms (RIC) obtained by GC–MS for *m*/*z* 85 and *m*/*z* 192, as well as GC–MS/MS-MRM for improved selectivity and sensitivity in identifying hopanes and steranes; (2) analysis of inorganic biomarkers using energy-dispersive X-ray spectroscopy (EDX); (3) comprehensive analysis of the polar fraction using UHR MS; and (4) multivariate analysis to assess sample similarity or dissimilarity. For forensic purposes, two possibilities were considered: (i) the oil sample in the drum is related to samples from the Sergipe–Alagoas Basin; (ii) the sample in the drum is related to samples involved in the spill case. This protocol was designed to improve the reliability of geochemical origin and source identification of spilled oil, addressing challenges faced by conventional oil spill identification methods.

## 2 Materials and Methods

### 2.1 Sample Collection

Three oil samples related to the spill were provided to the Biomass Petroleum and Energy Research Group (PEB) by the Brazilian Institute of the Environment and Renewable Natural Resources (IBAMA) and the Mass Spectrometry Research Group (PEM) of the Federal Rural University of Pernambuco. Two of these samples were collected from different points along the coast of Sergipe, while the last was collected from the coast of Pernambuco.

The other four samples, also suspected of being involved in the case, refer to the main oil-producing streams in the Sergipe–Alagoas basin (SE–AL) and were supplied by an oil company operating in the basin. The last sample used in this study was collected inside a closed drum found on the Sergipe coast at the same time as the oils associated with the spill, as mentioned earlier; the image of the drum can be found in the Supporting Information (Figure S1). Regarding the samples provided by the company, information about the location and date of collection was considered confidential. Information on the location of the sample, the code, and the date on which it was acquired is summarized in Table 1.

**Table 1:** Detailed Information on Oils from the Spill, Samples of Oils from the SE–AL Basin, and the Source Oil Drum

code	state	local	date
sample 1	Sergipe	10°43′57.05″S; 36°50′29.02″W	09/28/2019
sample 2	Sergipe	10°52′49.04″S; 36°58′39.01″W	09/28/2019
sample 3	Pernambuco	08°23′16.00″S; 34°57′55.00″W	10/20/2019
sample T	Sergipe	10°48′48.00″S; 36°55′11.00″W	09/28/2019
sample A	Sergipe	off-shore (Sergipe)	confidential
sample B	Alagoas	on-shore (Alagoas)	confidential
sample C	Sergipe	off-shore (Sergipe)	confidential
sample D	Sergipe	on-shore (Sergipe)	confidential

### 2.2 Preparation of Spill Oil Samples

Following the international guidelines published by the International Tanker Oil Pollution Federation (ITOPF), the samples were collected with as few solids as possible.^24^ However, the presence of sand in the oil was still observed, as shown in Figure S2, so it was decided that the samples would be cleaned before analysis. To do this, approximately 2 g of the oil/sand mixture was diluted in 5 mL of toluene and centrifuged to settle the solid particles. The supernatant was stored in a separating funnel to remove residual water. This process was repeated until the solvent became colorless, with a maximum of five repetitions. The organic component was then separated using a separating funnel. Toluene was removed using a rotary evaporator, resulting in a brown oily residue. The extracted oil was stored in an amber bottle at 8 °C to minimize any alterations until subsequent analysis.

### 2.3 Separation of the SARA Fractions from the Oils Investigated

To reduce the complexity of the oils investigated (oils from the SE–AL basin, the drum, and the spill), SARA fractionation was performed. The method employed was the same as that described by Carregosa et al. (2023).^25^ Briefly, after precipitating the asphaltene fraction with *n*-hexane, the maltene fraction was collected and taken to the rotary evaporator for determination of its content and subsequent SAR fractionation (saturates, aromatics, and resins). To obtain the SAR fractions, 10 mg of the maltenes was diluted in 100 μL of *n*-hexane, and then, using a glass pipet, the resulting solution was transferred to the top of a chromatographic column (12.5 cm long and 0.6 cm in diameter), filled with 500 mg of G60 flash silica, and sieved to 115 mesh. The silica was previously fired in a muffle furnace at 400 °C for 4 h and then mixed with a quantity of distilled water equivalent to 5% (m/m) of the mass of silica used. The silica was packed into the chromatographic column using *n*-hexane. Five mL borosilicate glass vials were used to collect the SAR fractions. The mobile phases used were 3 mL of *n*-hexane, 2 mL of *n*-hexane/dichloromethane (7:3), and 2 mL of toluene/methanol (1:1). The fractions collected were dried under a nitrogen flow.

### 2.4 Characterization of Nonpolar Biomarkers by GC–MS-RIC and GC–MS/MS

The fractions of saturates from the oils from the SE–AL basin, the drum, and the spill were diluted to a concentration of 10 mg mL^–1^ in dichloromethane. The solutions of the saturated fractions of the oil samples from the SE–AL basin, the drum, and the spill were analyzed in a GC–MS system with a triple quadrupole mass analyzer (GCMS-TQ8040—Shimadzu) and electron ionization (EI), provided by the Multiuser Chemistry Laboratory Center (CLQM). The capillary column used was SH-RTX5SilMS (Crossbond, composed of 5% diphenyl and 95% dimethylsilyphenylene siloxane, 30 m long, 0.25 mm i.d., 0.25 μm film thickness, Restek, USA). The samples were injected in splitless mode, a volume of 1 μL was used, and the injector temperature was set at 300 °C. The oven temperature was programmed from 70 to 325 °C at 3 °C min^–1^. Helium (99.999% purity) was used as the carrier gas at a constant flow rate of 1.0 mL min^–1^. The mass spectrometer was operated in electron ionization mode (EI) at 70 eV. The ion source temperature was 280 °C, and the interface temperature was 290 °C. The MS was calibrated daily by autotuning with perfluorotributylamine (PFTBA), and the chromatograms were acquired in full scan mode (mass range acquisition was performed from *m*/*z* 45 to 500). The total chromatographic run time was 85 min.

The compounds were identified by comparing retention times and elution patterns with GC–MS results and from literature refs 3, 26–28 . For GC–MS–RIC, the extracted ions were *m*/*z* 85 for *n*-alkanes^29^ and *m*/*z* 192 for the analysis of anthracenes and thiophenes.^30,31^ Equations 1 and 2 present the general formula recommended in the CEN standard.^32^ It is used for the diagnostic ratios where *A* and *B* are the area values for each biomarker from the same GC–MS injection.

1 $$DR=\frac{A}{B}$$

2 $$DR=\frac{A}{A+B}$$

For the GC–MS/MS analyses, argon (99.999% purity) was used as the collision gas, and the collision energy for each of the transitions was 12 eV. The transitions monitored are shown in Table 2.

**Table 2:** Transitions (Precursor Ions and Product Ions) Used in the Investigation and Identification of Steranes and Hopanes in MRM Mode

compounds	precursor ion (*m*/*z*)	product ion (*m*/*z*)
steranes C_27_	372	217
steranes C_28_	386	217
steranes C_29_	400	217
steranes C_30_	414	217
trisnorhopanes C_27_	370	191
hopanes C_29_	398	191
hopanes C_30_	412	191
homohopanes C_31_	426	191

### 2.5 Determination of the Nickel, Vanadium, and Total Sulfur Content of the Oils Investigated by Energy-Dispersive X-ray Fluorescence Spectrometry

The contents of nickel, vanadium, and sulfur in the investigated oils were determined using a Shimadzu energy-dispersive X-ray fluorescence spectrometer, model EDX-720/800HS, provided by CLQM. The equipment’s working range was between atoms S–K (15 kV) with a total analysis time of 100 s, operating in quantitative mode. The efficiency of the equipment was tested by comparing the results of analyzing a reference sample composed of Cr (18.395%), Mn (1.709%), Fe (70.718%), Ni (8.655%), Cu (0.278%), and Mo (0.245%). To determine the sulfur content, ASTM D4294 was used as a ref 33. As for the nickel and vanadium contents, ASTM D8252-19e1 was adopted as a ref 34. All these elements were determined in duplicates.

### 2.6 Characterization of Neutral-Basic Polar Compounds by H-ESI­(+)-FT-Orbitrap MS for the Investigated Oils

The analysis of the resin fractions of the oils from the SE–AL basin, the drum, and the spill was conducted on an Exactive HCD Plus system (Thermo Scientific, Bremen, Germany), provided by CLQM. The sample introduction method was by direct infusion using a 500 μL syringe (Thermo Scientific, NJ, USA) at a flow rate of 35 μL min^–1^. The ionization source used was heated electrospray (H-ESI). The sample was dissolved in a mixture of toluene/methanol (1:3 v/v), yielding a solution of 150 μg mL^–1^. The analysis conditions for the H-ESI mode were positive polarity (+), an equipment resolution of 140 000 at *m*/*z* 200, a spray voltage of 4.0 kV, heating of the vaporization region to 110 °C, a capillary temperature of 320 °C, sheath gas and auxiliary gas at 7 au (arbitrary unit), and S-lens at 70 rf. The acquisition of mass spectra was performed in the *m*/*z* range of 100–1200. A total of 100 μ scans were accumulated to obtain the final average mass spectrum.

The software determined the exact mass, inferred molecular formulas, indicated the assignment error for each molecular formula, provided double bond equivalent (DBE) values, and calculated the total abundance of ions used in the MS ratio proposed. Confirmation of peak attribution is considered to be agreement between the experimental and theoretical *m*/*z* values, together with the isotopic pattern. For the elemental compositions observed simultaneously in the sample and blank spectra, the final intensities were determined as the difference between the intensities of the sample and blank spectra.

The obtained results were processed by using an advanced data processing approach. The Xcalibur Qual Browser program was used to assign the molecular formulas to the ions. At this stage, up to 10 possible molecular formulas were accounted for each *m*/*z* with an error of less than 3 ppm. The criteria for assigning elemental compositions were ^13^C_0–1_, ^12^C_5–100_, ^1^H_5–200_, ^14^N_0–4_, ^16^O_0–8_, and ^32^S_0–3_. The list of ions was then transported to Microsoft Excel, where, using an advanced processing algorithm developed by the Petroleum and Energy from Biomass (PEB) research group, the most probable molecular formula was selected. To this end, the ^13^C isotopologue standard and Kendrick mass defects (±0.001 tolerance) were analyzed to confirm the accuracy of the elemental composition assignments.^35^ Mass peaks relevant to the isotopic distributions were identified and deleted. Only ions with a signal-to-noise ratio greater than 3 were considered.

### 2.7 Statistical Treatment for GC–MS/MS-MRM and UHR MS Data

For univariate statistical analysis, the method proposed by Kienhuis et al. (2016) based on the critical difference (CD) was used.^5^ Briefly, the individual critical difference is calculated by multiplying the repeatability limit at a 95% confidence level (14%) by the ratio between the difference between the values obtained for the diagnostic ratio evaluated (DR *z*) for oils A and B and the average of these values, as shown in eq 2.

3 $$CD=\frac{\left|DR\;z\;of\;A-DR\;z\;of\;B\right|}{\mathrm{X̅}\;DR\;z\;of\;A\;\text{and}\;B}\times r_{95\%}$$

The prerogative of the test is that if the critical difference is not exceeded, then there is no doubt that the diagnostic ratios are identical. In other words, for two reasons to be corresponding (identical), the absolute difference (AD) between two ratios must not be greater than the value of the critical difference for the same ratio.^5^ Therefore, in an investigative context, the argument that two oils are equal is strengthened by the greater number of diagnostic ratios whose absolute difference does not exceed the value of the critical difference at a 95% confidence level.

For the multivariate data analysis, principal component analysis (PCA) and hierarchical clustering analysis (HCA) were used to identify clustering trends among the oil samples from the SE–AL basin, the drum, and the spill. For PCA, the RStudio 3.6.1 program was used. Regarding the data obtained by GC–MS/MS-MRM, initially, the biomarker ratios of the samples (DP samples) were organized in matrices (6 × 23) and (6 × 36), respectively. In these, the objects are in the rows (DP samples) and the variables (biomarker ratios) are in the columns. The preprocessing used was data autoscaling, which consisted of subtracting the variable from the mean of the set and dividing the result by the standard deviation of the set.

For the mass spectra of the oil samples obtained by H-ESI­(+)-FT-Orbitrap MS, the data was organized into matrices for Kendrick nominal mass (KNM), 8 × 8425, and Kendrick defect mass (KDM), 8 × 464, where the objects are in the rows (DP samples) and the variables (*m*/*z* intensities) are in the columns. For this set, the preprocessing used was to center on the mean. In both cases, for data obtained by GC and UHR MS, the standard operating procedures of the mdatools package were followed to perform the treatment via PCA and HCA. The codes used are available at: https://cran.r-project.org/web/packages/mdatools/mdatools.pdf.

## 3 Results and Discussion

### 3.1 Evaluation of Similarity between Spill Samples and Suspected Oils by GC–MS-RIC (*m*/*z* 85)

Chemical fingerprinting of the *n*-alkane fraction of an oil is one of the main approaches to distinguishing and differentiating the sources of unknown oil and refined products associated with spills in the environment.^36^ In general, the first stage for investigating oil–oil correlations is GC-FID. However, the TICCs in GC–MS are equivalent and therefore provide the same information about the sample profiles. In addition, GC–MS allows the chromatogram to be reconstructed from a specific ion (RIC), reducing background effects and increasing the intensity of compounds containing the selected ion.^37^

Regarding the oil profiles of Sergipe–Alagoas, Figure 1, similar distributions were observed, with bimodal patterns skewed toward medium-to-short-chain *n*-alkanes, except for oil “C” (sample C), for which the distribution showed a bimodal pattern skewed toward long-chain *n*-alkanes. Bimodal distributions, especially those with a predominance of the *n*-C_23_ to *n*-C_31_ range, are generally associated with waxes from terrigenous higher plants, as observed for oil C.^15^

**Figure 1:**
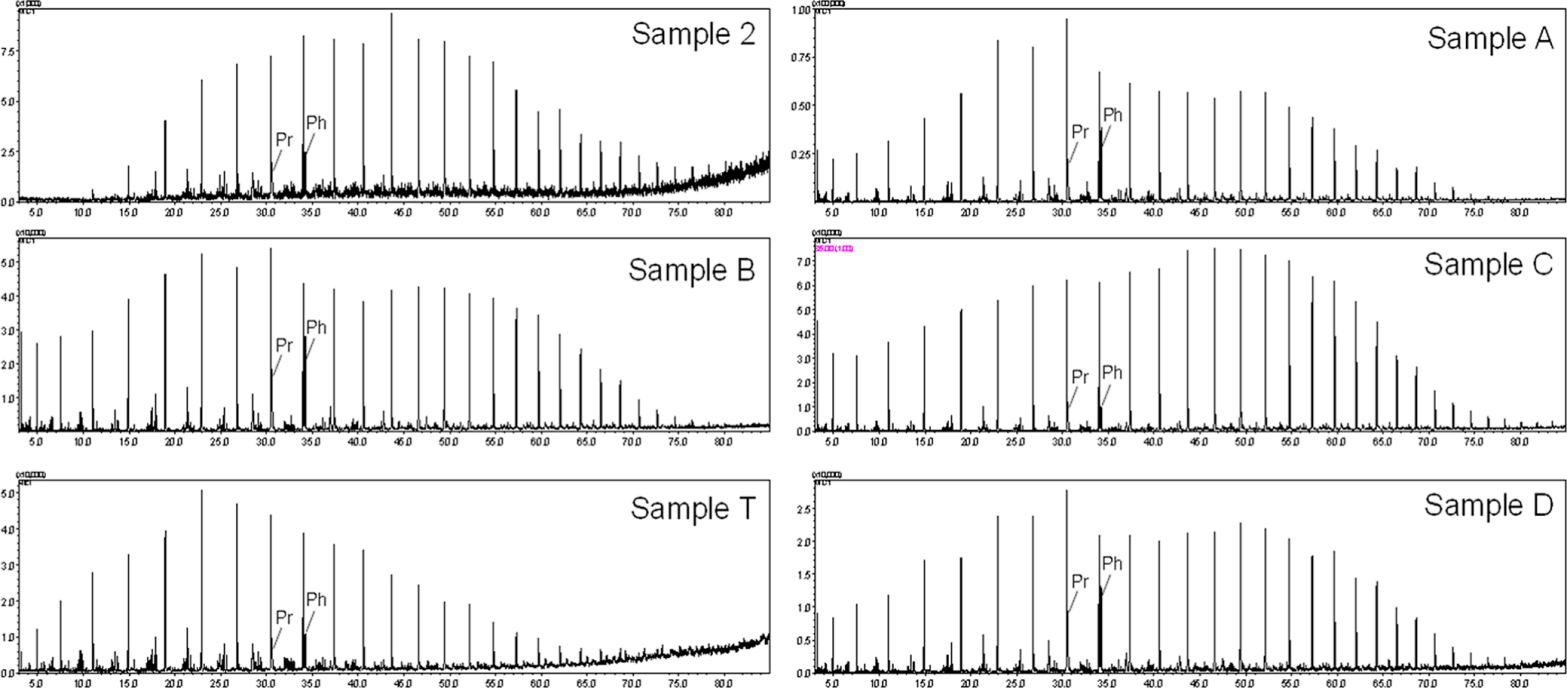
Comparative chromatograms obtained by GC–MS, reconstructed using ion *m*/*z* 85. Profiles correspond to suspected oils (samples A, B, C, D, and T) and to the spill oils (represented by sample 2).

For oils “A”, “B”, and “D” (sample A, sample B, and sample D, respectively), the skewed distribution toward lighter paraffins indicates a contribution from both terrigenous and marine origins, with a greater contribution from marine biomass.^15,28^ Regarding the oil from the drum (sample T), a different profile was observed from the others, showing a unimodal distribution with a predominance of short-chain compounds. This distribution is strongly characteristic of the input of organic matter from algae.^15,28^

In a previous investigation published by our group, which used the same samples used in this work, it was determined that sample 1, sample 2, and sample 3 are equivalent.^13^ Thus, comparing the paraffinic compound profiles of the oils from the spill (represented by sample 2) and those produced in Sergipe, it was observed that, while the spill samples showed a paraffinic compound pattern ranging from *n*-C_12–13_ to *n*-C_33–34_, with a bimodal distribution skewed toward medium- and long-chain *n*-alkanes, the profiles of the crude oils from the SE–AL basin showed a wider range of paraffinic compounds, varying from *n*-C_7_ to *n*-C_37_. Although they showed bimodal behavior, they did not share similarities with the spill samples. Similarly, the profile of the oil in the drum also showed no similarity with either the SE–AL basin samples or the spill samples when assessing the distribution of *n*-alkanes.

Considering that tocopherols can also be a precursor of pristane in ancient sediments and zooplankton and archaebacteria can also be possible precursors of Pr and Ph, the phytyl side chain can be cleaved, generating pristane (Pr) and phytane (Ph) preferentially in oxidizing and reducing environments, respectively. Hence, the Pr/Ph ratio can be used to evaluate the redox environment. Liu et al. (2021) suggested a relatively oxic condition during the depositional process, characterized by a transitional environment with an oxic condition and more terrestrial inputs.^38^ In their research, they found values that were similar to those previously published by our group for the set of oil samples from the spill. As for the pristane/phytane (Pr/Ph ratios of the spill oils), values of 1.71 for sample 1, 1.65 for sample 2, and 1.88 for sample 3 were determined. These values indicate that the reservoir rock was deposited under suboxic conditions.^13,38^

### 3.2 Evaluation of the Presence or Absence of Thermal Alteration on Oil Samples from the Spill, SE–AL Basin, and the Drum by GC–MS–RIC (*m*/*z* 192)

Previous studies on the classification of oils landed on Brazilian beaches, by evaluating the distributions of C1-phenanthrene/anthracene isomers and C1-dibenzothiophenes, have indicated that the oil from the spill is some thermally altered material.^39^ Based on this, the reconstructed chromatogram of the *m*/*z* 192 ion, Figure 2, was constructed to visualize the distribution of methylphenanthrene and, consequently, categorization of the oils from the spill, the drum, and the SE–AL basin.

**Figure 2:**
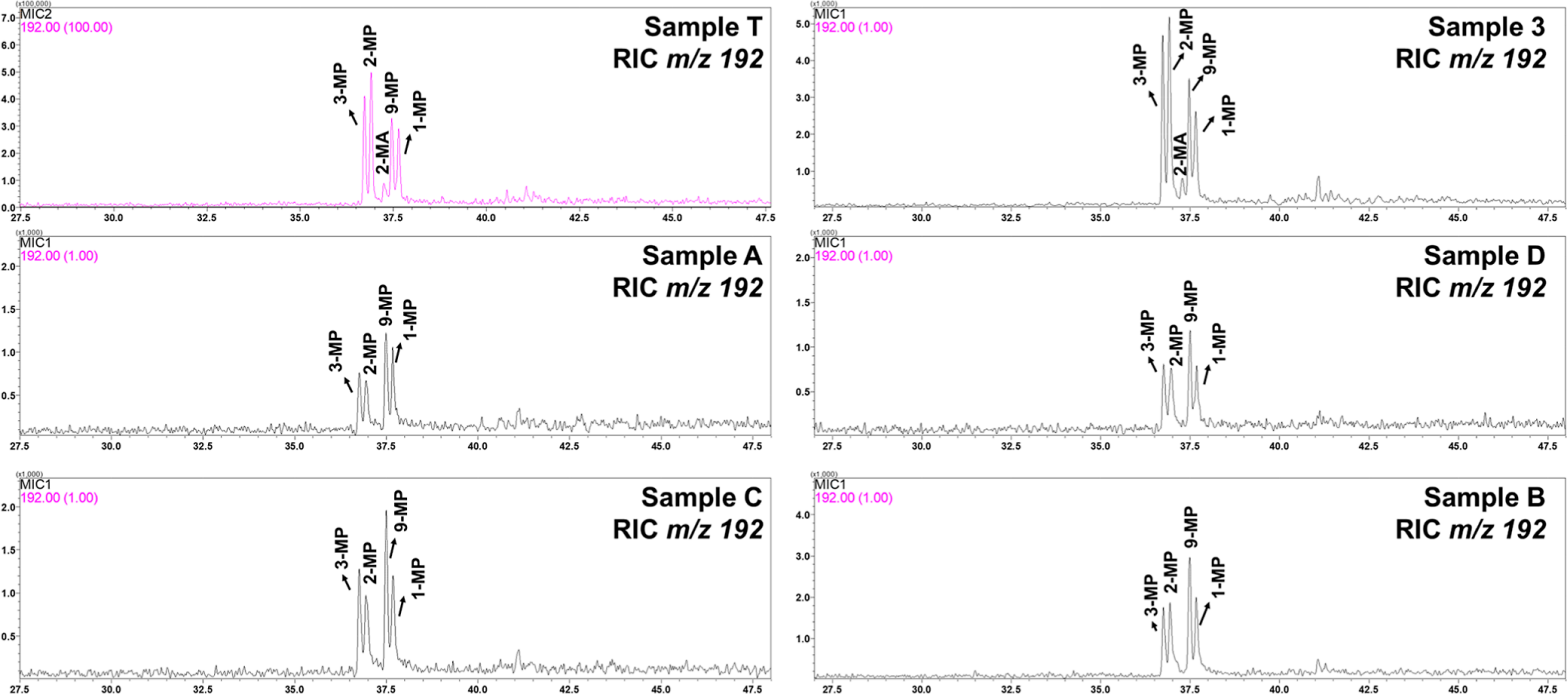
Comparison of methylphenanthrene and 2-methylanthracene profiles (*m*/*z* 192) in spill, drum, and SE–AL basin samples.

For thermally treated crude oils, the compounds 2- and 3-methylphenanthrene (2-MP and 3-MP, respectively), which are more thermally stable than 9/4- and 1-methylphenanthrene (9-MP and 1-MP, respectively), show a higher peak height than 9-MP and 1-MP.^40^ In addition, the biomarker 2-methylanthracene (2-MA) was detected above the noise of the chromatogram only in the samples from the drum (sample T) and the spill (represented by sample 3). The presence of this compound is seen in trace concentrations in crude oils and derivatives, but its concentration is high in thermally treated oils.^39^ Third, the ratio of biomarkers obtained from the ratio of 2-methylanthracene to total methylphenanthrene was 0.04 and 0.05 for sample 3 and sample T, respectively, within the range of 0.04–0.09 in modern heavy fuel oils containing cracked materials.^39^ This result corroborates that described by Reddy et al. (2022), who stated that such findings indicate that the oil from the Brazil spill contains refined materials and not just virgin crude oil.

### 3.3 Evaluation of Similarity between Samples from the Spill, the SE–AL Basin Oils, and the Drum by GC–MS/MS-MRM

The geochemical characterization of crude oils using a triple quadrupole mass analyzer operating in MRM mode allows the resolution of various coelutions in various transitions referring to steranes, hopanes, and PAHs.^41^ The individual peak of each biomarker was confirmed based on the retention time and reference chromatograms,^3,26–28^ and these are available in the Supporting Information, Figures S3–S12. The relationship between the names of each biomarker and its retention time can be seen in Table 3.

**Table 3:** Sterane and Hopane Biomarker Compounds Identified by GC–MS/MS-MRM

retention time (min)	peak number	diagnostic ratio code	compound name
62.501	1	C_27_ααR	5α(H),14α(H),17α(H)-cholestane (20R)
62.092	2	C_27_ββS	5α(H),14β(H),17β(H)-cholestane (20S)
61.836	3	C_27_ββR	5α(H),14β(H),17β(H)-cholestane (20R)
61.650	4	C_27_ααS	5α(H),14α(H),17α(H)-cholestane (20S)
59.859	5	DIA_27_αβR	13α(H),17β(H)-diacholestane (20R)
59.484	6	DIA_27_αβS	13α(H),17β(H)-diacholestane (20S)
58.838	7	DIA_27_βαR	13β(H),17α(H)-diacholestane (20R)
58.083	8	DIA_27_βαS	13β(H),17α(H)-diacholestane (20S)
64.852	9	C_28_ααR	5α(H),14α(H),17α(H)-ergostane (20R)
64.344	10	C_28_ααS	5α(H),14β(H),17β(H)-ergostane (20S)
64.090	11	C_28_ββR	5α(H),14β(H),17β(H)-ergostane (20R)
63.879	12	C_28_ββS	5α(H),14α(H),17α(H)-ergostane(20S)
62.149	13	DIA_28_αβR	13α(H),17β(H)-diaergostane (20R)
61.553	14	DIA_28_αβS	13α(H),17β(H)-diaergostane (20S)
61.006	15	DIA_28_βαR	13β(H),17α(H)-diaergostane (20R)
60.101	16	DIA_28_βαS	13β(H),17α(H)-diaergostane (20S)
66.778	17	C_29_ααR	5α(H),14α(H),17α(H)-estigmastane (20R)
66.115	18	C_29_ββS	5α(H),14β(H),17β(H)-estigmastane (20S)
65.919	19	C_29_ββR	5α(H),14β(H),17β(H)-estigmastane (20R)
65.578	20	C_29_ααS	5α(H),14α(H),17α(H)-estigmastane (20S)
62.776	23	DIA_29_βαR	13β(H),17α(H)-diaestimagtane (20R)
61.932	24	DIA_29_βαS	13β(H),17α(H)-diaestimagtane (20S)
68.496	25	ISO30R	5α(H),14α(H),17α(H)-24-isopropilcholestane (20R)
67.434	28	ISO30S	5α(H),14α(H),17α(H)-24-isopropilcholestane (20S)
63.056	40	Ts	18α(H)-22,29,30-trisnorhopane (Ts)
64.002	42	Tm	17α(H)-22,29,30-trisnorhopane (Tm)
66.901	45	H29	17α(H),21β(H)-30-norhopane (C29Hop)
67.347	48	C30Diahop	C3017α(H)-diahopane
68.680	49	H30	17α(H),21 β (H)-hopane (C30Hop)
69.547	52	MOR30	17β(H),21α(H)-hopane (C30baHop)
71.377	53	GAM	gammacerane
70.754	55	H31S	17α(H),21β(H)-homohopane (22S)
71.004	56	H31R	17α(H),21β(H)-homohopane (22R)
72.398	61	H32S	17α(H),21β(H)-bishomohopane (22S)
72.727	62	H32R	17α(H),21β(H)-bishomohopane (22R)
68.344	65	OL	18α(H)-oleanane + 18β(H)-oleanane

To assess the similarity, 35 biomarker ratios were calculated. The results are shown in Table 4. The geochemical similarity assessment between the spill samples (represented by sample 2) and the oil samples from the SE–AL basin and the oil from the drum was also performed after scaling the data, Table 4.

**Table 4:** Biomarker Diagnostic Ratios for Spill, Drum, and Basin Oils Determined by GC–MS/MS-MRM

diagnostic ratios	sample 2	sample T	sample A	sample B	sample C	sample D
Ts/Tm	0.888	0.731	0.832	0.910	1.121	0.992
Ts/(Ts + Tm)	0.470	0.422	0.454	0.476	0.528	0.498
H29/H30	0.473	1.044	0.226	0.291	0.215	0.323
MOR30/H30	0.015	0.040	0.055	0.062	0.057	0.081
GAM/H30	0.035	0.039	0.130	0.270	0.210	0.231
H31S/H31R	1.544	1.693	1.647	1.427	1.284	1.516
H32S/H32R	1.710	1.588	1.430	1.371	1.578	1.413
C30Diahop/H30	0.046	0.035	0.095	0.085	0.109	0.099
H31R/H30	0.311	0.301	0.177	0.205	0.121	0.165
steranes/hopanes^a^	1.288	0.622	2.811	3.535	1.610	2.854
C_29_ααS/(C_29_ααS + C_29_ααR)	0.522	0.434	0.417	0.485	0.283	0.371
C_27_ββ(R + S)/C_29_ββ(R + S)	0.919	0.546	0.605	0.553	0.685	0.431
C_27_ααS/C_27_ααR	1.425	0.933	0.883	1.093	0.526	0.879
C_28_ααS/C_28_ααR	1.138	1.135	0.585	0.652	0.030	0.311
C_29_ααS/C_29_ααS	1.091	0.767	0.717	0.943	0.395	0.590
ISO30S/ISO30S + ISO30R	0.220	0.550	0.277	0.442	0.097	0.434
DIA27αβ/DIA27βα + Dia27αβ	0.041	0.270	0.233	0.230	0.099	0.221
C_27_ααS/C_27_ααR	1.425	0.933	0.883	1.093	0.526	0.879
C_27_ββS/C_27_ββR	1.031	0.999	1.166	0.911	0.734	0.863
C_27_αα(S + R)/C_27_αα(S + R)+C_27_ββ(S + R)	0.485	0.535	0.623	0.673	0.896	0.788
DIA28αβ(S + R)/DIA28βα(S + R)+Dia28αβ (S + R)	0.172	0.216	0.294	0.241	0.247	0.314
C_28_ααS/C_28_ααR	1.138	1.135	0.585	0.652	0.030	0.311
C_28_ββS/C_28_ββR	0.091	0.101	0.138	0.184	1.755	0.346
C_28_αα(S + R)/C_28_αα(S + R)+C_28_ββ(S + R)	0.560	0.581	0.555	0.602	0.908	0.713
DIA29S/DIA29R	1.882	1.397	1.282	1.219	0.000	1.245
C_29_ααS/C_29_ααR	1.091	0.767	0.717	0.943	0.395	0.590
C_29_ββS/C_29_ββR	0.550	0.567	0.558	0.537	0.135	0.313
C_29_αα(S + R)/C_29_αα(S + R) + C_29_ββ(S + R)	0.473	0.474	0.514	0.576	0.691	0.633
H32S/(H32R + H32S)	0.631	0.614	0.588	0.578	0.612	0.586
C_29_ββ(S + R)/(C_29_ββ(S + R) + C_29_αα(S + R))	0.527	0.526	0.486	0.424	0.309	0.367
OL/(OL + H30)	0.160	0.051	0.000	0.000	0.000	0.000
GAM/(GAM + H30)	0.034	0.038	0.115	0.213	0.173	0.188
diasteranes/steranes^b^	0.175	0.288	0.245	0.573	0.195	0.267
% C_27_ααR^c^	30%	31%	37%	34%	56%	35%
% C_28_ααR^d^	33%	20%	21%	22%	20%	21%
% C_29_ααR^e^	37%	49%	42%	44%	23%	44%

a [(C_27_–C_29_αα (S + R) + C_27_–C_29_ββ (S + R))/(C_29_–C_33_αβ hopanes [S + R])].

b [(DIAC27βα–DIAC29βα (S + R))/(C_27_–C_29_αα (S + R) + C_27_–C_29_ββ (S + R))].

c C_27_ααR/(C_27_–C_29_)­ααR.

d C_28_ααR/(C_27_–C_29_)­ααR.

e C_29_ααR/(C_27_–C_29_)­ααR.

For PCA, the Euclidean distance can be used to assess the similarity between vectors, i.e., between the values of a variable in different observations. The smaller the Euclidean distance between two vectors, the more similar they are. When evaluating the PCA score graph (Figure 3), it was seen that just two principal components already explained 82.3% of the variance in the data, with PC 1 accounting for 60.07% and PC 2 accounting for 22.29%.

**Figure 3:**
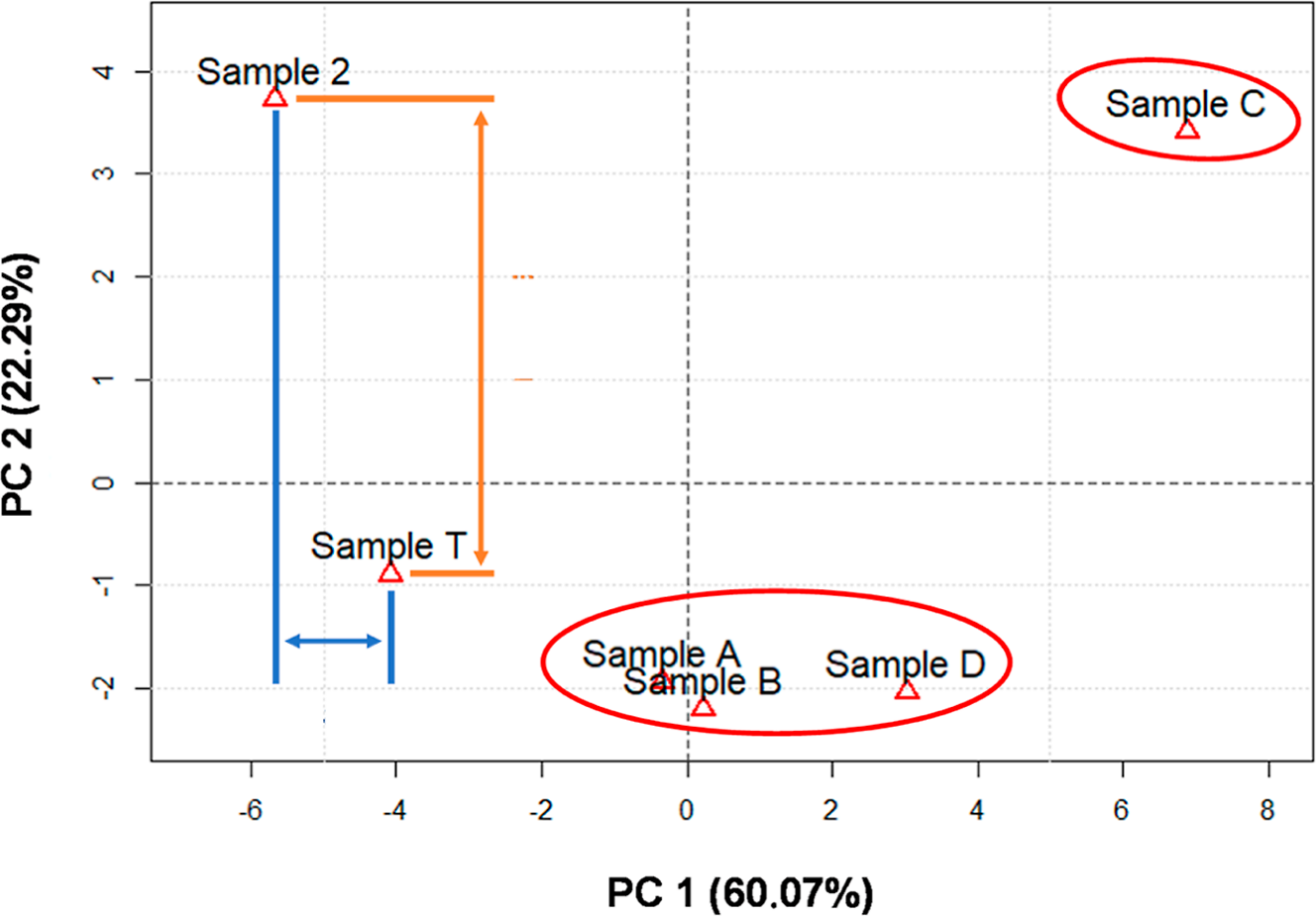
Principal component analysis of hopane and sterane distributions in spill, drum, and SE–AL basin oils (GC–MS/MS-MRM) for source identification.

The score plot showed that the ratios obtained by GC–MS/MS-MRM, in a multivariate PCA-type treatment, were able to group and separate the Sergipe samples (samples A–D) from the Alagoas sample (sample C). Furthermore, considering that PC 1 explains the similarity/dissimilarity of 60% of the selected variables, the Euclidean distance observed on the PC 1 axis between samples sample T and sample 2 is indicative of a correlation between the samples.

The weight graph of PC 1 presented in Figure 4 shows that the separation on the “*x*” axis is mainly caused by the biomarker ratios H31/H30 and H31/H30 e C_29_ββS/C_29_ββR (positively), and the ratios Ts/Tm, Ts/(Ts + Tm), C_27_αα­(S + R)/C_27_αα­(S + R) + C_27_ββ­(S + R), C_28_ββS/C_28_ββR, and % C_27_ααR (negatively), which can subsequently be grouped into three geochemical parameters: organic matter input, thermal evolution, and depositional paleoenvironment.

**Figure 4:**
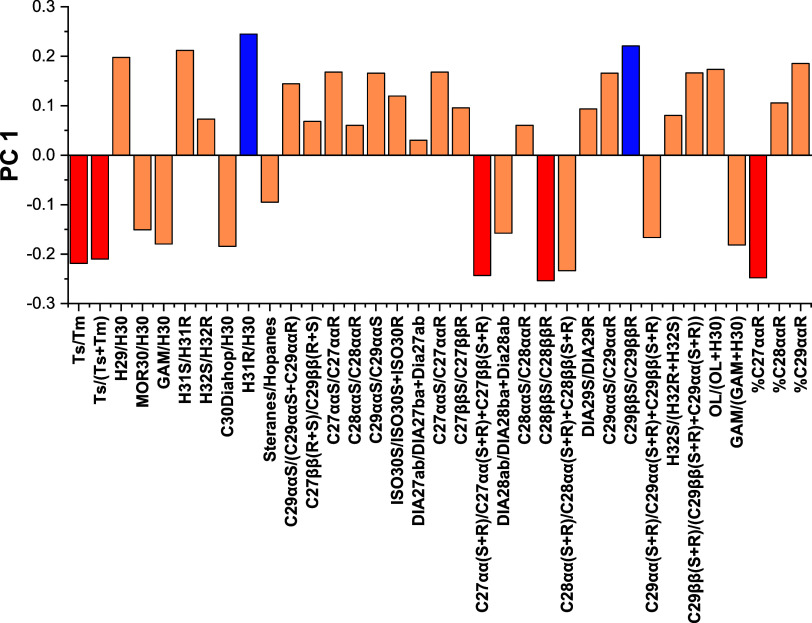
PC 1 loadings plot of GC–MS/MS-MRM diagnostic ratios for source differentiation of spill, drum, and SE–AL basin oils.

Based on the loadings observed in PC 1, considering the contribution of organic matter to the oil samples from the SE–AL basin, the drum, and the spill, the ratio of biomarkers % C_27_ααR indicated that sample T and sample 2 have similar contributions of organic matter, with a predominance of terrigenous organic matter and a lower contribution of marine organic matter from the contribution of zooplankton. As for thermal evolution, the indicators C_29_ββS/C_29_ββR, Ts/Tm, Ts/(Ts + Tm), and C_28_ββS/C_28_ββR showed that sample 2 and sample T had similar thermal evolution processes. Comparing sample T and sample 2 with samples from the SE–AL basin, it was demonstrated that samples A, B, C, and D had a greater degree of thermal evolution.

In terms of the depositional paleoenvironment, the H31/H30 indicator was useful in discriminating between marine and lacustrine depositional environments. Unlike crude oils from rocks of lacustrine origin, oils from marine shale, carbonate rocks, and rocks of marl origin generally show H31R/H30 values greater than 0.25. The values obtained for this ratio (Table 4) ranged from 0.12 to 0.31, with sample 2 and sample T showing values higher than 0.25. Therefore, in contrast to the crude oils from the SE–AL basin, the oils from the spill and the drum came from a marine carbonate, shale, or marl environment.^2,3^ Therefore, based on the PC 1 loadings, sample T and sample 2 share a similar geochemical background, and they are both unrelated to the SE–AL crude oil samples.

Regarding the loading plot for PC 2 shown in Figure 5, the discrimination of the samples is based on the separation on the “*y*” axis. It was observed that this was mainly caused by the ratios DIA27αβ/DIA27βα + Dia27αβ, DIA28αβ/DIA28βα + Dia28αβ, and % C29ααR (positively) and negatively by the biomarker ratios H32S/H32R, C27ββ­(R + S)/C29ββ­(R + S), H32S/(H32R + H32S), and Oleanane index (% OI), calculated by OL/(OL + H30).^42^ The diacholestane (DIA27) and diaestigmastane (DIA28) ratios were tabulated in this material for oil–oil correlation purposes. In contrast, the others mentioned could be grouped into two geochemical parameters: thermal evolution and the input of organic matter.

**Figure 5:**
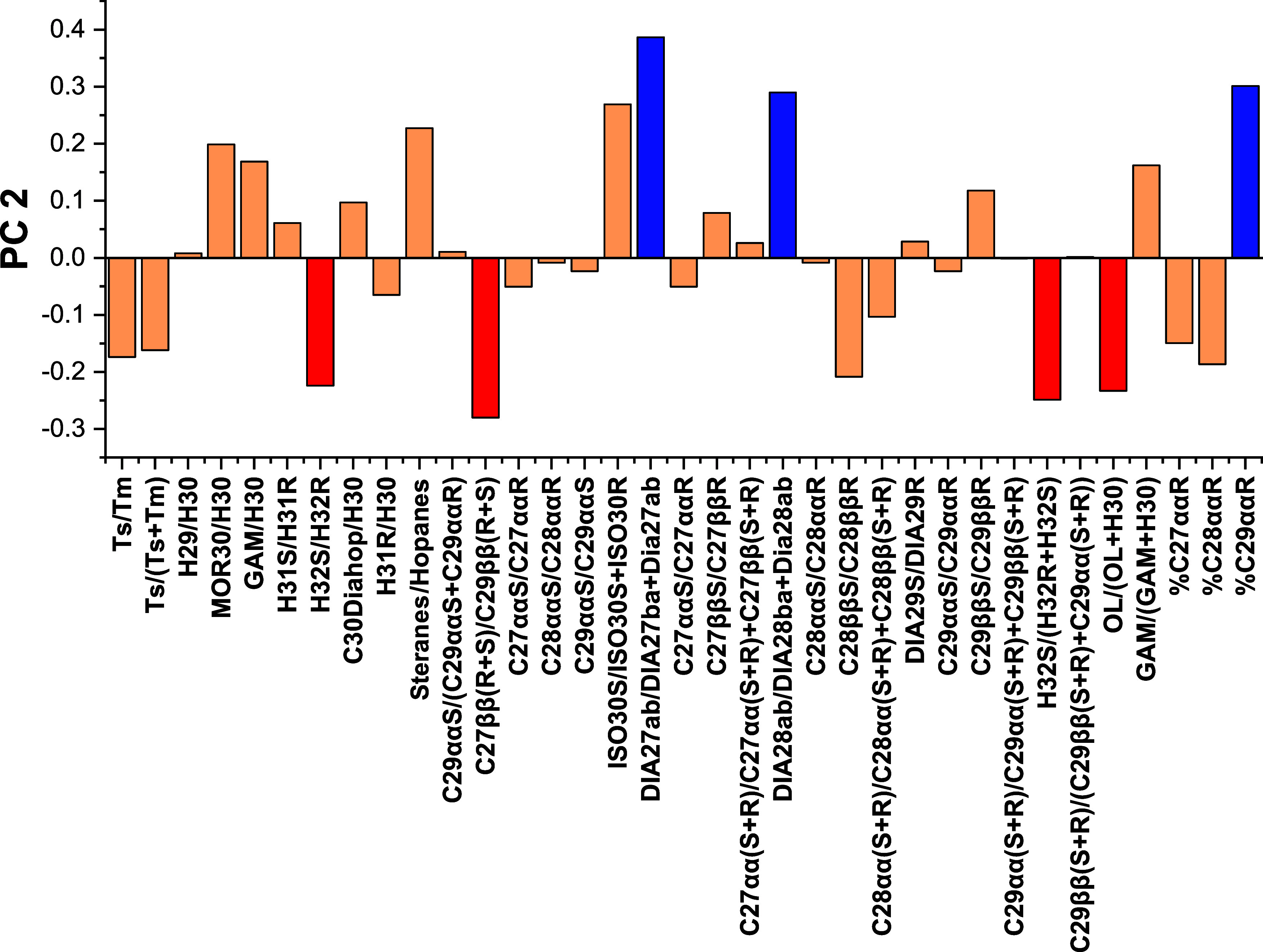
PC 2 loadings plot of GC–MS/MS-MRM diagnostic ratios for source differentiation of spill, drum, and SE–AL basin oils.

During thermal maturation, the values of the H32S/H32R + H32S ratio increase from 0, when there is only the R isomer, to approximately 0.6 (0.57–0.62), when a balance is reached between the concentrations of the isomers present, indicating maturity equilibrium.^3^ In this sense, the differentiation of thermal evolution, via PC 2, by the indicator mentioned earlier, indicates that the oil samples collected on the beaches of the states of Pernambuco and Sergipe, represented by sample 2, as well as samples T and C, are close to the equilibrium range of maturity, which means they are more thermally evolved. However, for this marker, there is no clear distinction between the degrees of thermal evolution between oil collected on the beaches (sample 2) and oil collected inside the drum (sample T). In contrast, the H32S/H32R marker shows a more evident difference between the two samples. This biomarker ratio also indicates the degree of thermal evolution due to the conversion of the R isomer into the S isomer as the oil evolves thermally.^28^ Thus, it indicates that sample 2 comes from a more thermally evolved oil than the others investigated.

In terms of organic matter input, it is observed via PC 2 that the indicators % C_29_ααR and % OI are also responsible for separating the investigated samples. Regarding Oleanane, which is present in crude oils and rock extracts, it belongs to a different class of biomarker, which is a highly specific biomarker.^1^ This biomarker, which has two isomers, 18α-(H)-oleanane and 18β-(H)-oleanane, is found only in rocks and oils from the tertiary and cretaceous (<130 million years) and is highly specific to inputs from angiosperm plants.^43^ Besides, biodegradation processes and water washing, which are the most critical postaccumulation processes in reservoirs, do not alter the structure of either 18α­(H)-oleanane or 18β­(H)-oleanane. This fact makes this biomarker robust and hence very useful in oil–oil and oil–source rock correlation studies.^42^ Therefore, oil sample T and sample 2 shared similar contributions of organic matter and formation age solely on the basis of this specific biomarker.

#### 3.3.1 Univariate Statistical Comparison of Sterane and Hopane Diagnostic Ratios Obtained by GC–MS/MS-MRM for the Drum and Spill Oils

Since the oil sample contained in the drum (sample T) showed a higher correlation with the oil spill sample (represented by sample 2), these two were evaluated according to the diagnostic ratios of biomarkers obtained by GC–MS/MS-MRM, adapting the method proposed by CEN and used by Stout et al. (2016).^5,40^ Based on the diagnostic ratios, sample 2 and sample T showed greater similarities for the biomarkers belonging to the hopane class than among the sterane-type compounds.

To quantify the level of similarity, a univariate statistical treatment based on relative standard deviation (RSD) and critical difference was performed. The prerogative of the test is that if the critical difference is exceeded, then there will be no doubt that the biomarker ratios are identical. In other words, for two ratios to be corresponding (matching), the absolute difference (AD) between the two ratios must not exceed the critical difference (CD) value for the same ratio. Thus, in an investigative context, the argument that two oils are equal is strengthened by the greater the number of biomarker ratios whose absolute difference exceeds the critical difference value at a 95% confidence level.^5^

For this evaluation, “YES” means that the absolute difference is less than the critical difference or that the relative standard deviation (RSD %) is less than 10%, and therefore, the diagnostic ratios are related. As for “NO”, it means that the previous rule was not followed, and then, they are not related. The results are summarized in Table 5.

**Table 5:** Univariate Comparison of Diagnostic Ratios Determined for the Spill Samples and the Oil from the Drum by GC–MS/MS-MRM

diagnostic ratio	sample 2	sample T	average	RSD %	absolute difference (AD) (%)	critical difference (CD) (%)	CD < AD	RSD < 10%
Ts/Tm	0.888	0.731	0.810	14	16	11	no	no
Ts/(Ts + Tm)	0.470	0.422	0.446	8	5	6	yes	yes
H29/H30	0.473	1.044	0.758	53	57	11	no	no
MOR30/H30	0.015	0.040	0.028	63	2	0	no	no
GAM/H30	0.035	0.039	0.037	8	0	1	yes	yes
H31S/H31R	1.544	1.693	1.619	7	15	23	yes	yes
H32S/H32R	1.710	1.588	1.649	5	12	23	yes	yes
C30Diahop/H30	0.046	0.035	0.041	19	1	1	no	no
H31R/H30	0.311	0.301	0.306	2	1	4	yes	yes
Steranes/Hopanes	1.288	0.622	0.955	49	67	13	no	no
C_29_ααS/(C_29_ααS + C_29_ααR)	0.522	0.434	0.478	13	9	7	no	no
C_27_ββ(R + S)/C_29_ββ(R + S)	0.919	0.546	0.733	36	37	10	no	no
C_27_ααS/C_27_ααR	1.425	0.933	1.179	30	49	17	no	no
C_28_ααS/C_28_ααR	1.138	1.135	1.137	0	0	16	yes	yes
C_29_ααS/C_29_ααS	1.091	0.767	0.929	25	32	13	no	no
ISO30S/ISO30S + ISO30R	0.220	0.550	0.385	61	33	5	no	no
DIA_27_αβ/DIA_27_βα + Dia_27_αβ	0.041	0.270	0.156	104	23	2	no	no
C_27_ααS/C_27_ααR	1.425	0.933	1.179	30	49	17	no	no
C_27_ββS/C_27_ββR	1.031	0.999	1.015	2	3	14	yes	yes
C_27_αα(S + R)/C_27_αα(S + R)+C_27_ββ(S + R)	0.485	0.535	0.510	7	5	7	yes	yes
DIA_28_αβ/DIA_28_βα + Dia_28_αβ	0.172	0.216	0.194	16	4	3	no	no
C_28_ααS/C_28_ααR	1.138	1.135	1.137	0	0	16	yes	yes
C_28_ββS/C_28_ββR	0.091	0.101	0.096	7	1	1	yes	yes
C_28_αα(S + R)/C_28_αα(S + R) + C_28_ββ(S + R)	0.560	0.581	0.571	3	2	8	yes	yes
DIA_29_S/DIA_29_R	1.882	1.397	1.640	21	48	23	no	no
C_29_ααS/C_29_ααR	1.091	0.767	0.929	25	32	13	no	no
C_29_ββS/C_29_ββR	0.550	0.567	0.558	2	2	8	yes	yes
H32S/(H32R + H32S)	0.631	0.614	0.622	2	2	9	yes	yes
C_29_ββ(S + R)/(C_29_ββ(S + R)+C_29_αα(S + R))	0.527	0.526	0.527	0	0	7	yes	yes
% OI = OL/(OL + H30)	16.0%	5.1%	10.6%	73	11	1	no	no
% GI = GAM/(GAM + H30)	3.4%	3.8%	3.6%	7	0	1	yes	yes
% C_27_	30.5%	31.3%	30.9%	2	1	4	yes	yes
% C_28_	32.8%	19.7%	26.3%	35	13	4	no	no
% C_29_	36.7%	49.0%	42.9%	20	12	6	no	no
diasteranes/steranes	0.175	0.245	0.210	23	7	3	no	no

The biomarkers C_29_ββ­(S + R)/(C_29_ββ­(S + R) + C_29_αα­(S + R)), C_28_ααS/C_28_ααR, Ts/(Ts + Tm), H32S/(H32R + H32S), H31S/H31R, and H32S/H32R indicate that the samples from the drum and the spill originated from source rocks that reached the maturity equilibrium for hydrocarbon generation.^44,45^ Moreover, the Gammacerane index (% GI) between 3% and 4% indicates variations in stratification conditions in the water column, which is characteristic of marine or deltaic limestone rocks.^28,46^ Corroborating this information, the H31/H30 ratio with values of 0.301 and 0.311 for the drum and spill samples, respectively, indicated that the oils from the spill and the drum originated in a marine carbonate environment, shale, or marl.

Regarding the input of organic matter, although it is observed that the % C_27_ ratio is positively correlated, indicating a similar contribution of marine biomass from zooplankton, it is noted that the largest contribution was from the C_29_-sterane. Therefore, these are samples with a mixed organic matter contribution, with a greater contribution of terrestrial organic matter. The oleanane index (% OI), which is an important biomarker derived from terrestrial plants, indicated an organic matter contribution related to the sediments from the middle/late Cretaceous period.^28,45^

Considering all the data contained in Table 5, it was observed that out of the 35 biomarker ratios evaluated, 16 positively corresponding (45.7%). Han et al. (2020) had already shown that there is a difference in the quantification of oil biomarkers by SIM and MRM, as MRM proved to be more precise due to the decrease in the signal-to-noise ratio and the increase in selectivity.^10^ In this sense, considering the results of the univariate treatment performed with the data acquired by MRM, it was observed that the similarity relationship became inconclusive, unlike the conclusion published by the local authorities, which found that the similarity relationship between the spill sample and the drum was nonexistent.

### 3.4 Characterization of Total Sulfur Content and Inorganic Biomarkers by EDX for the Oils from the Drum, the SE–AL Basin, and the Oil Spill

A layer concerning the geochemical characterization of inorganic biomarkers and total sulfur content was added to the protocol due to the inconclusiveness obtained through the analysis of apolar organic biomarkers. The ratios between some trace elements are used as indicators for the deposition environment of source rocks; moreover, they have been used to investigate correlations between oil samples in forensic approaches. Moreover, even under severe weathering conditions, they do not exhibit significant variations, making them, thus, similar to steranes and hopanes in terms of resistance and weathering in the marine environment.^1,47–49^

Among these elements, the proportions of nickel (Ni) and vanadium (V) porphyrins are used as source parameters in oil–oil and source rock–oil correlations. Vanadium and nickel are the main metals in petroleum, but they are not part of the precursors originating from living organisms. These metals enter the porphyrin structure through complexation during the initial stage of diagenesis, and the depositional environment is the primary influence on their respective relative proportions.^40,50,51^ Furthermore, the V/Ni or V/(Ni + V) ratios together with the sulfur content (% S) allow for the determination of paleo-redox or biodegradation conditions.^50^

The nickel and vanadium concentrations, as well as the sulfur content, were determined for all the samples in duplicate, including the results of the samples from the main crude oil producing fields in SE–AL, and are shown in Table 6. When comparing the spill samples, it was observed that they corroborate what has been discussed so far; i.e., that they come from the same source, since their biomarker ratios are equivalent, including the V/Ni and V/(Ni + V) ratios, as well as the mass percentage of sulfur (% S).

**Table 6:** Assessment of V/Ni Ratios and Sulfur Content by EDX for Oil Source Correlation and Depositional Environment Analysis^a^

	diagnostic ratios and trace elements content				
sample	V (ppm)	Ni (ppm)	V/Ni	V/(V + Ni)	% S (m/m)
sample 1	105.59 ± 1.89	14.10 ± 1.39	7.52 ± 7.52	0.88 ± 0.88	0.82 ± 0.01
sample 2	92.52 ± 0.68	12.07 ± 0.64	7.68 ± 0.35	0.88 ± 0.00	0.84 ± 0.02
sample 3	106.99 ± 1.09	15.10 ± 0.66	7.09 ± 0.24	0.88 ± 0.00	0.75 ± 0.00
sample T	111.34 ± 0.20	14.83 ± 1.07	7.53 ± 0.56	0.88 ± 0.01	0.71 ± 0.01
sample A	ND	ND	NC	NC	0.23 ± 0.01
sample B	ND	14.74 ± 0.48	NC	NC	0.45 ± 0.01
sample C	ND	11.78 ± 0.04	NC	NC	0.07 ± 0.10
sample D	ND	15.92 ± 1.29	NC	NC	0.69 ± 0.01

a *ND = not detected; *NC = not calculated.

The concentration ranges of the spill oils for the elements vanadium and nickel were 92.52–106.99 ppm and 12.07–15.10 ppm, respectively. Compared with the values determined for sample T, the nickel content is within the range obtained for the oil spill samples. However, in terms of vanadium content, sample T differs significantly from the spill samples. With respect to the sulfur content, the spill samples showed values between 0.75% and 0.84%; thus, sample T is also close to the highlighted range, showing a DPR of between 4 and 8% concerning the minimum and maximum values. It is worth noting that the samples from the SE–AL basin bear no resemblance to those from the spill or the oil sample contained in the drum.

Assessing the Lewan diagram, shown in Figure 6, by using the diagnostic ratio V/(V + Ni) and % S, revealed that the oils from the spill and the drum are located in zone 2 (V/(V + Ni) > 0.1 and <0.9; and S < 1%).^52^ The oils within this zone are associated with the deposition of marine-terrestrial organic matter in a hypoxic/suboxic environment,^28^ corroborating the diagnosis of the pristane over phytane ratio (Pr/Ph) obtained for the spill oils (1.75 ± 0.1).^13^ The oils from Sergipe were not grouped in the graph due to the impossibility of calculating the ratio in question, given the absence of the vanadium element in their oils. Based on the trace elements and sulfur content, the oils from the spill and the drum share similar formation conditions in terms of the depositional paleoenvironment and the contribution of organic matter.

**Figure 6:**
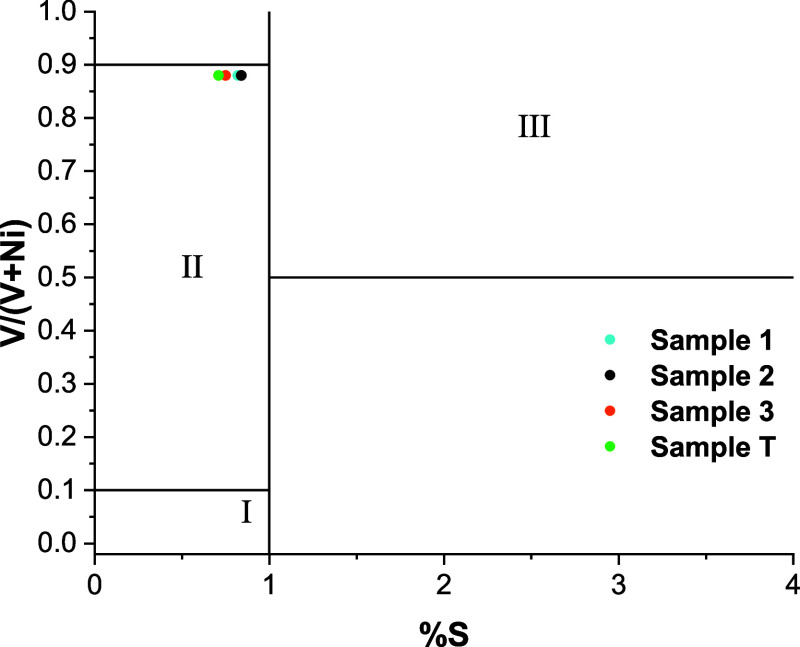
Correlation diagram between the V/(V + Ni) ratio and sulfur content for the classification of the depositional paleoenvironment of oils. Note: I (terrestrial oxic), II (marine-terrestrial hypoxic-suboxic), and III (marine carbonate anoxic).

### 3.5 Evaluation of Similarity/Dissimilarity of Oil Samples from the SE–AL Basin, the Drum and the Spill by H-ESI­(+)-FT-Orbitrap MS

To determine the applicability of the H-ESI­(+)-FT–Orbitrap MS technique for oil correlation, data quality control was initially carried out.^53^ In terms of data quality, between 12 and 17,000 molecular ions were detected, of which ∼7800 molecular ions (∼42% on average) were assigned molecular formulas. Within this set, approximately 3000 molecular formulas (∼50% on average) had a signal-to-noise ratio greater than 3 (S/N > 3) with errors of less than 3 ppm. Concerning the error associated with determining the molecular composition, it was observed that more than 50% of the data consisted of molecular formulas with an assignment error of less than ± 1 ppm (∼1700 molecular formulas). Table S1 summarizes all of this information.

#### 3.5.1 Multivariate Analysis for Similarity Determination among the Oil Samples from the SE–AL Basin, the Drum, and the Spill Based on the Data Obtained by H-ESI­(+)-FT-Orbitrap MS

After quality control of the chemical formulas assigned to the molecular ions detected in the oil samples from the SE–AL basin, the drum, and the spill, the data set was preprocessed, and subsequent multivariate analysis via HCA and PCA was carried out to assess similarity/dissimilarity relationships. The statistical analysis was performed considering each Kendrick’s nominal mass (KNM) with its absolute abundance in each mass spectrum as a different variable.^54^ For similarity analysis via HCA, the criteria adopted were the square of the Euclidean distance and the unweighted pair group method with subtraction of the arithmetic mean, also known as average linkage clustering. In addition, the vertical scale was normalized to 100%.^55^ Thus, the lower the percentage of dissimilarity observed, the greater the similarity between the compared samples.

In the HCA plot, Figure 7, considering the maximum dissimilarity for the set to be 100%, three groups were formed from the exploratory analysis of the data, referring to the distribution of KNM for the basic polar compounds of the oil samples from the SE–AL basin, the drum, and the spill. One group consisted of sample C; the other consisted of the different samples from the SE–AL basin (samples A, B, and D), and the other consisted of sample T and the spill samples.

**Figure 7:**
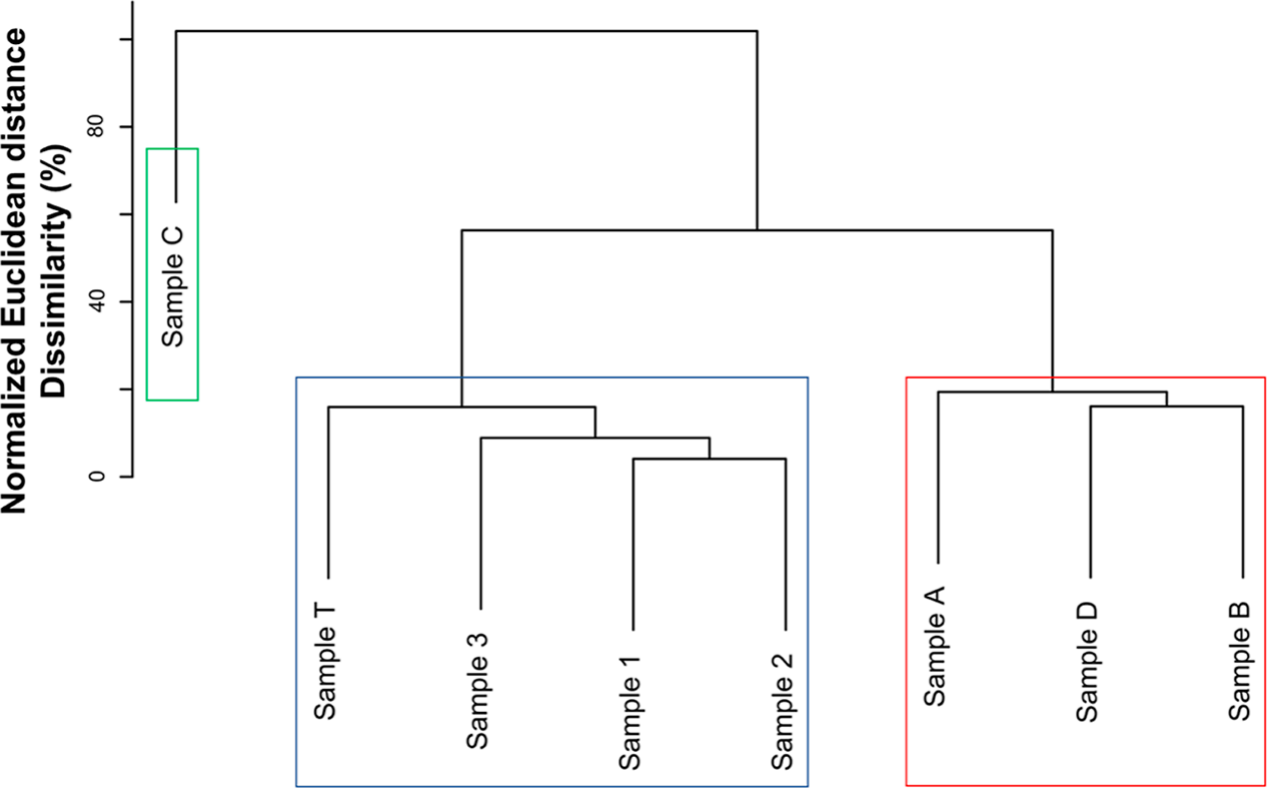
Geochemical clustering of samples based on UHR MS data obtained by H-ESI­(+)-FT-Orbitrap MS for differentiating spill, drum, and SE–AL basin oils.

Regarding the similarity and dissimilarity relationships verified by HCA, the spill samples share a similarity relationship with the drum sample (sample T). Within this group, when considering the second hierarchical level, the samples found in Sergipe (samples 1 and 2) were separated from the ones found in Pernambuco (sample 3). And when considering the third hierarchical level in relation to the set of spill samples, sample T was grouped with a dissimilarity value close to ∼17%, which means ∼83% of similarity considering the normalized Euclidean distance. Furthermore, the multivariate analysis showed that the dissimilarity between the spill and drum samples in relation to those from SE–AL was ∼60% for samples A, D, and B and 100% for sample C.

From the multivariate analysis by PCA, Figure 8, in which the two axes correspond to the first two principal components (PC 1 and PC 2), PC 1 accounted for around 90% of the explained variance and PC 2 accounted for around 5%. Therefore, the total variance of the two components accounts for 95% of the variation in the original data.

**Figure 8:**
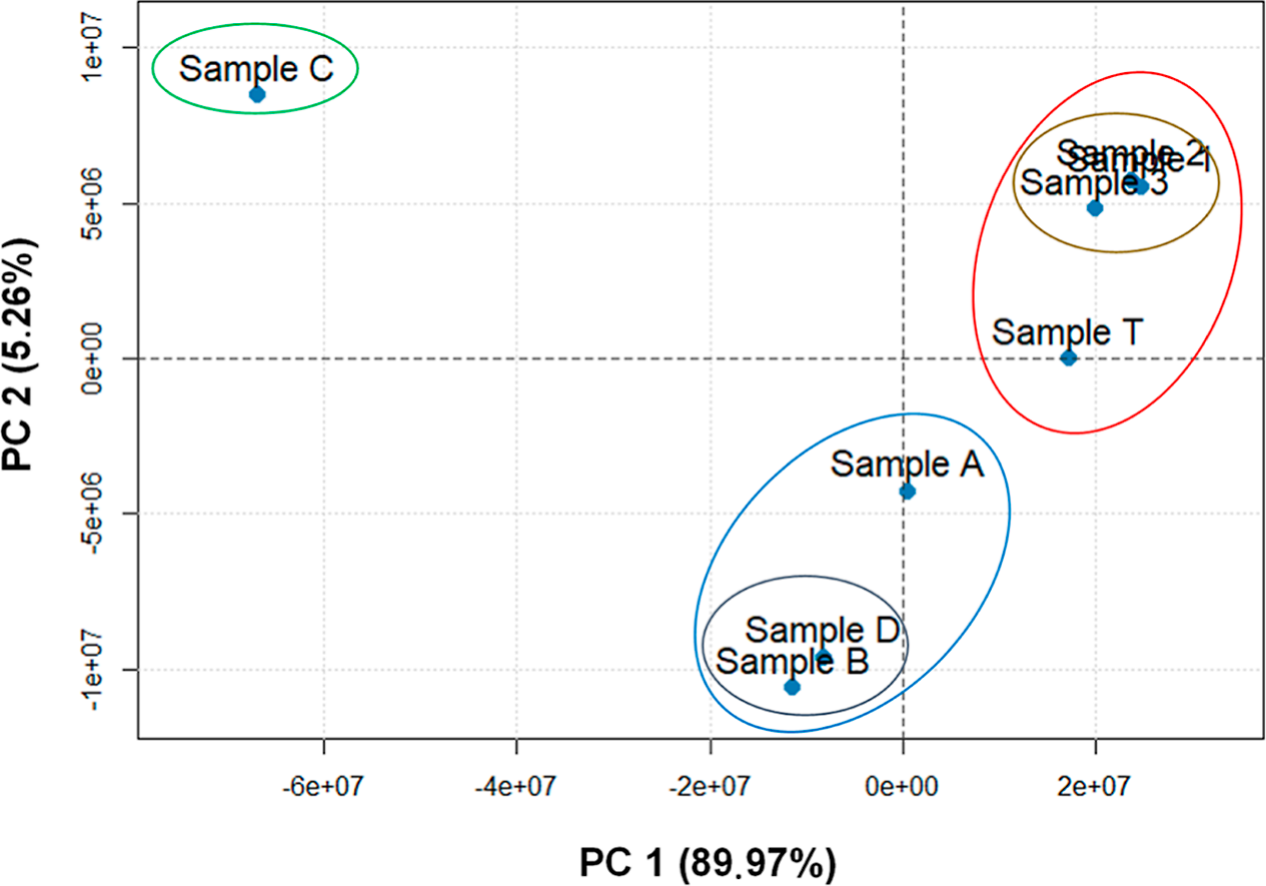
Score plot based on the KNM distributions of the oil samples from the SE–AL basin, the drum, and the spill obtained by H-ESI­(+)-FT-Orbitrap MS.

PCA and HCA produce similar results in terms of separation and clustering. The results shown in the score graph for the H-ESI­(+)-FT-Orbitrap MS data corroborate the separation observed in the score graphs obtained for the GC–MS/MS data. Based on the PCA score plot, sample B and sample D have a neutral and basic molecular composition similar to that of sample A. However, sample C, which is known to be from the same basin, showed no similarity for this set of compounds. This can be explained by the fact that sample C comes from the Alagoa portion of the SE–AL basin. Although sample B was extracted in the same geographical area as samples A and D, it was shown in a different quadrant of the PCA score plot. These differences may be associated with the organic matter input of these samples, which may have had different contributions, as shown for the distribution of regular steranes presented in Table 4.^54^

The multivariate analysis based on the data obtained by H-ESI­(+)-FT-Orbitrap MS proved to be effective in determining the similarity between the spill and drum samples, especially considering the *X*-axis (PC 1), which alone accounts for almost 90% of the variables. The small distance observed on the PC 1 axis is strong evidence that the samples have similar polar basic compounds. To determine the chemical characteristics of the samples and identify their differences, molecular-level distribution plots were generated for all of the samples involved in this case.

#### 3.5.2 Characterization of the Oil Samples from the Spill, the SE–AL Basin, and the Drum by UHR MS

The analysis of crude oil by UHR MS is one of the main challenges, as thousands of ions are detected in a single crude file, and therefore, the individual analysis of each detected compound, as is done with GC–MS and GC–MS/MS data, would take a lot of time and effort. Therefore, the best way to visualize the data sets obtained by this technique is via graphical analysis using petroleomics. In general, the data processing program operates based on an algorithm capable of combining elements previously selected from the periodic table, commonly carbon (C), oxygen (O), nitrogen (N), and sulfur (S), into molecular formulas containing errors of less than 5 ppm in relation to the experimental mass/charge. Based on the assignment of highly accurate molecular formulas, according to their respective elemental compositions, C_*c*_H_*h*_N_*n*_O_*o*_S_*s*_, the compounds are grouped. Thus, for example, an ion assigned the molecular formula “C_16_H_32_O_2_” will be grouped molecularly with the heteroatomic group O_2_, together with the molecule “C_7_H_6_O_2_.” After grouping, diagrams and histograms are plotted to visualize the molecular distribution better.^56–59^ The most common plots for data visualization are histograms of groups, diagrams of DBE vs carbon number, van Krevelen diagrams, and Kendrick mass diagrams.^53^

When evaluating the histogram of groups of the spill samples, Figure S13, some groups were not detected for all of the oils, such as the O_1_S_1_, O_2_S_1_, and O_1_ groups. However, the majority was the same in all the samples (N_1_, N_1_S_1_, O_3,_ O_1_N_1_S_1_, and O_1_N_1_). This result is consistent with those described by Reddy et al. (2022),^39^ who also detected all groups, except group O_3_, when they analyzed samples from the spill in other regions of the northeastern coast by ESI­(+)-FT-ICR MS. Sample 3 was selected as the representative for comparison with the samples under investigation. This approach enabled a more accurate comparison between the samples, providing valuable insights into their chemical composition and identifying potential sources of origin.

Based on the histogram of groups, Figure 9, sample T and sample 3 showed the same majority classes (N_1_, N_1_S_1_, O_3_, and O_1_N_1_). However, sample T showed an inversion of the second and third majority classes (N_1_S_1_ and O_3_) compared to that of the spill samples. In addition, among the classes related to the oils from the spill, only the classes of the O_1_N_1_S_1_ and the O_4_ classes were not detected for the drum sample. With respect to the samples from the SE–AL basin, sample T showed no similarities in terms of the distribution of groups observed by the histogram; the classes in common were: N_1_, O_3_, and O_1_N_1_.

**Figure 9:**
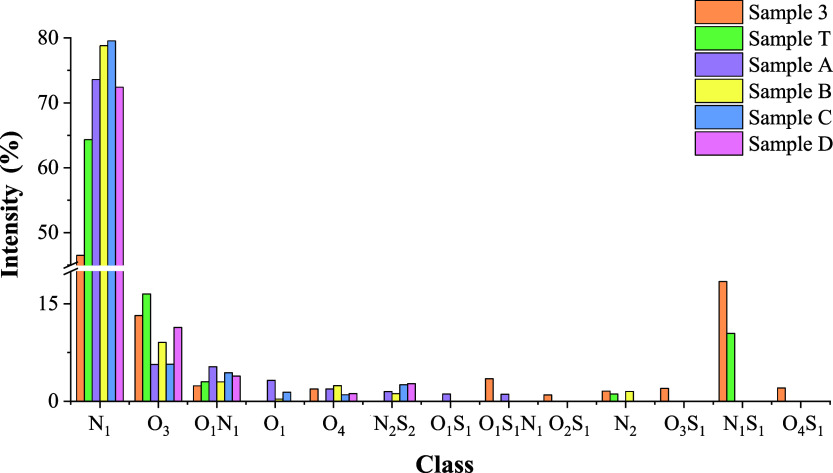
Histogram of groups of the oil samples from the SE–AL basin, the drum, and the spill (represented by sample 3) obtained by H-ESI­(+)-FT-Orbitrap MS.

Another approach used to determine the correlation between the data obtained by H-ESI­(+)-FT-Orbitrap MS was a comparison of Kendrick diagrams. This technique is based on the idea that compounds with the same composition (N, O, S) and the same number of DBE (number of rings and/or double bonds), but different numbers of CH_2_ units, will be different in the Kendrick diagram and identified as members of a homologous series. However, members of a homologous series will have the same Kendrick mass defect (KMD) that is unique to that series.^60^

Kendrick diagrams are used to identify molecular distribution patterns and are therefore useful tools for detecting molecular alterations caused by contamination or adulteration of samples. Furthermore, from these diagrams, it is possible to classify compounds according to their mass and degree of unsaturation.^61^ As it is understood that the profile observed in a Kendrick diagram is unique to each sample, its use as an oil–oil correlation tool can be more efficient than other ways of visualizing UHR MS data. Thus, the Kendrick plots were generated from the data obtained by H-ESI­(+)-FT-Orbitrap MS and are shown in Figure 10.

**Figure 10:**
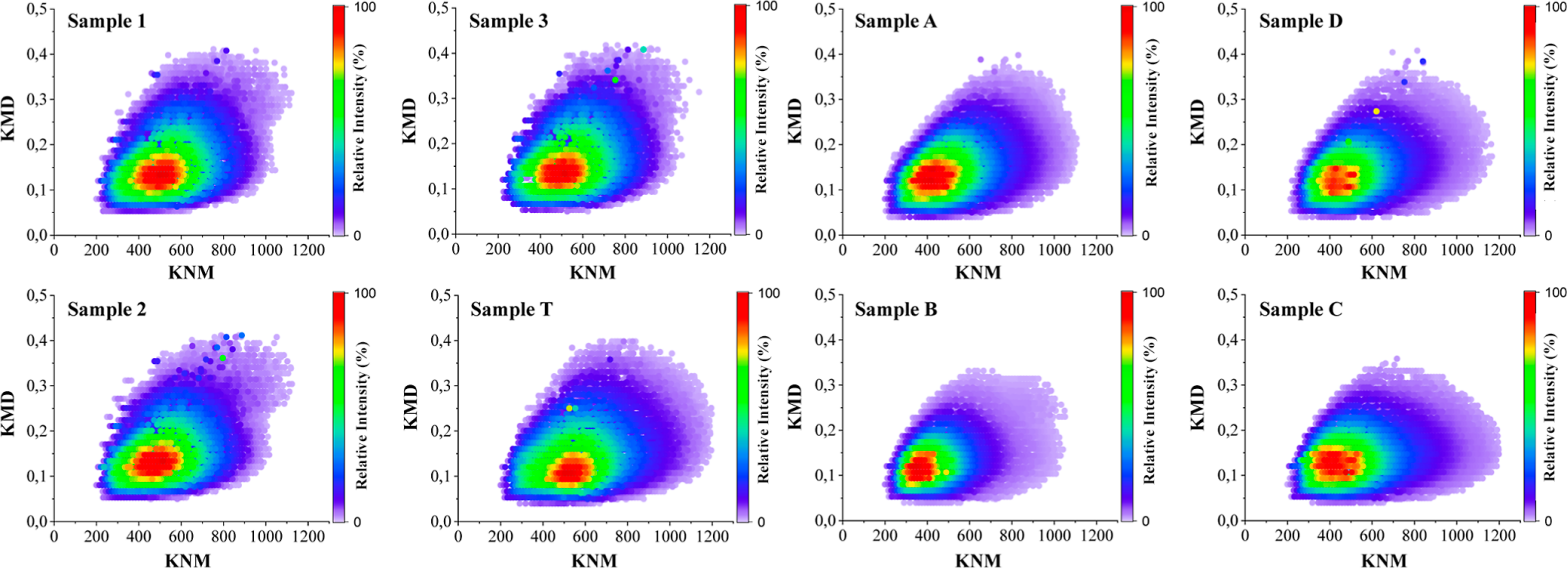
Polar basic compounds profile by Kendrick diagrams obtained by H-ESI­(+)-FT-Orbitrap MS for evaluation of similarity patterns among the oil samples from the SE–AL basin, the drum, and the spill.

The Kendrick distribution plots for the set of samples from the SE–AL basin showed different profiles for each sample. Sample A showed a distribution of KNM ranging from 200 to 1100 and KMD between 0.04 and 0.49, with the region of highest intensity found in the KNM range between 260 and 540 and KMD between 0.08 and 0.24. Sample B showed a distribution of KNM ranging from 200 to 1160 and a KMD between 0.040 and 0.46, with the region of highest intensity in the KNM range between 290 and 490 and a KMD between 0.09 and 0.15. Samples C and D showed the same range of KNM (between 200 and 1200) and KMD (0.04–0.47), but with different regions of greater intensity. Sample C showed the highest intensity in the KNM range between 305 and 560 and KMD between 0.08 and 0.40, while sample D showed the highest intensity in the KNM range between 330 and 550 and KMD between 0.93 and 0.16.

For the spill samples, it was found that samples 1, 2, and 3 showed a similar profile, with KNM ranging from 200 to 1180 and KMD between 0.039 and 0.46. In addition, the regions of highest intensity, highlighted by the red-orange zones (KNM between 390 and 640; KMD between 0.09 and 0.18), were also the same for this set of samples. In comparison to sample T, it was found that the distribution of KNM (200–1160) was similar to that of the oil spill samples. However, it was noted that this sample had lower aromaticity, as the KMD range is slightly lower (KMD between 0.04 and 0.40). The region of highest intensity for sample T was seen in the KNM range between 421 and 630 and the KMD range between 0.08 and 0.14, similar to that observed in the oil spill samples.

The comparative analysis of sample T and the SE–AL basin showed clear and significant differences. Sample A showed a lighter molecular mass at 180 Da when compared with the spill samples. For sample B, although the KNM and KMD distributions were similar, the most intense region was composed of molecules with a lighter molecular mass, with a difference of around 150 Da. In samples C and D, a difference of around 100 Da was also observed in the region of the most intense compounds. In general, sample T showed a more intense region composed of compounds with a higher molecular mass. These data are consistent with the previous techniques used and reinforce the hypothesis that the sample from the drum has a chemical composition different from that found in the SE–AL basin.

## 4 Conclusion

From the results discussed above, it was possible to unequivocally determine that the oils from the SE–AL basin could not have contributed to the disaster or had anything to do with the drum found concurrently with the spill. About the contents of the drum, the presence of the specific biomarker 18α­(H)-oleanane and the ∼45% similarity determined from the diagnostic ratios obtained by GC–MS/MS-MRM, as well as the similarity observed for the molecular composition of the polar compounds determined by H-ESI­(+)-FT-Orbitrap MS, both confirmed by multivariate statistical analysis, with emphasis on the HCA, which showed ∼83% similarity, show that the contents of the drum have geochemical aspects similar to those of the spill samples, especially concerning thermal evolution and the type of organic matter deposited.

Considering the date of the drum’s departure (17/02/2019) and the date on which the first oil slicks appeared on the northeastern coast, it can be said that the spill occurred between Feb/2019 and Aug/2019. This disregards the hypothesis that the oil came from a ship that sank in the middle of the last century. In addition, by using a more advanced framework in terms of geochemical characterization, this multilayer protocol proved necessary, since by using GC–MS–SIM-based methodology, the local authorities ruled out the relationship between the oil from the spill and the oil contained in the drum. By using more up-to-date methods and techniques, which go beyond the barriers of classic methodologies, new interpretations of the case have been raised since new arguments can be used to reintroduce the drum into federal investigations.

Finally, considering the proposal for an updated protocol for determining the correlation between spilled oil and suspected oil, this study suggests the following order of analysis.

1 Monitor the relationship between methylphenanthrene compounds and/or the presence of 2-methylanthracene to determine whether the samples have undergone heat treatment.
2 If it is confirmed that there has been alteration through heat treatment, then it is necessary to proceed to the determination of apolar markers via GC–MS/MS-MRM;
3 If it is still inconclusive, submit the samples to characterization of inorganic markers by EDX or equivalent technique and determination of the molecular profile of polar compounds by UHR MS using electrospray in positive mode.
4 Apply chemometric treatment via PCA and HCA to highlight similarities/differences.

In this way, the number of arguments to be considered in legal scrutiny will be more effective as traditional and advanced methods of organic and inorganic characterization will support it.

## Supplementary Material



## Notes

Drum found on the coast of Sergipe in September 2019; physical aspect of the samples collected on the beaches of Sergipe; chromatogram obtained by GC/MS/MS-MRM monitoring the transition for the main crude oil biomarkers; quality control of the data obtained by H-ESI­(+)-FT-Orbitrap MS for the oil samples; and histogram of groups obtained by H-ESI­(+)-FT-Orbitrap MS for the oil spill samples (PDF)
