# Supplementary material for: Multilayered Forensic Protocol Based on In-Depth Mass Spectrometry Techniques for the Investigation of Suspicious Drums of Oils with the 2019–2022 Brazilian Oil Spill Disaster
Source: ACS Omega. 2025 Oct 1;10(40):47438–54. doi: 10.1021/acsomega.5c07056 (PMC12529171; doi:10.1021/acsomega.5c07056)

SUPPLEMENTARY MATERIAL

MULTILAYERED FORENSIC PROTOCOL BASED ON IN-DEPTH MASS SPECTROMETRY TECHNIQUES FOR THE INVESTIGATION OF SUSPICIOUS DRUMS OF OILS WITH THE 2019-2022 BRAZILIAN OIL SPILL DISASTER

Jhonattas Carvalho Carregosa[1], Mirele Santana Sá[1], Jandyson Machado Santos[3], Alberto Wisniewski Jr[1*]

[1] Petroleum and Energy from Biomass Research Group (PEB), Department of Chemistry, Federal University of Sergipe, Jardim Rosa Elze, São Cristóvão, SE, 49107-230, Brazil

[3] Rural Federal University of Pernambuco, Department of Chemistry, Dois irmãos, Recife, PE 52171-900, Brazil

* Corresponding author: Alberto Wisniewski Jr.

Phone: +55 79 31947045

E-mail: albertowj@academico.ufs.br

Petroleum and Energy from Biomass Research Group (PEB), Department of Chemistry, Federal University of Sergipe, Jardim Rosa Elze, São Cristóvão, SE, 49107-230, Brazil

Figure S1. Drum found on the coast of Sergipe in September 2019.

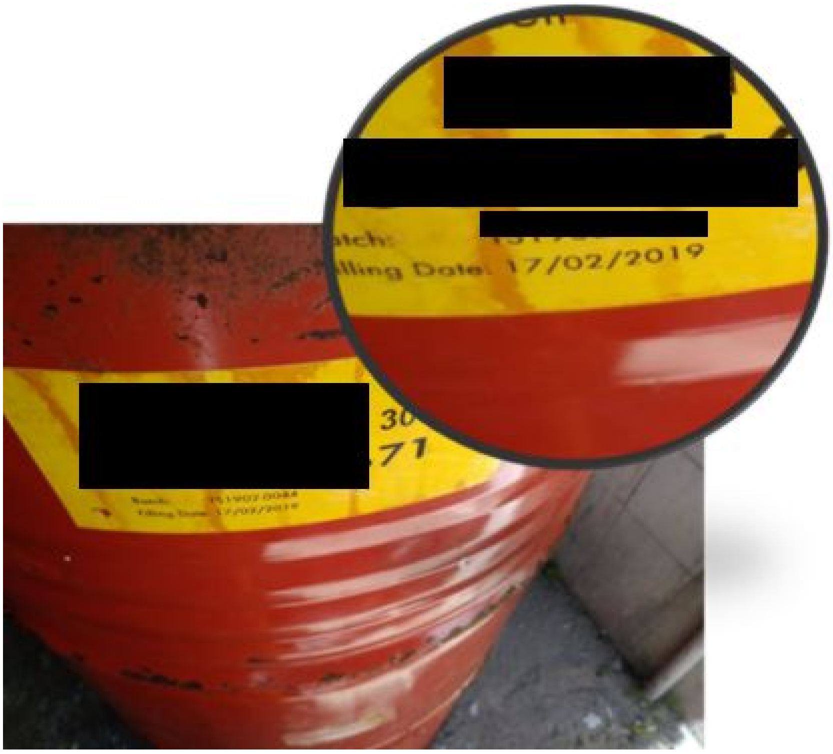

Figura 1. Physical aspect of the samples collected on the beaches of Sergipe.

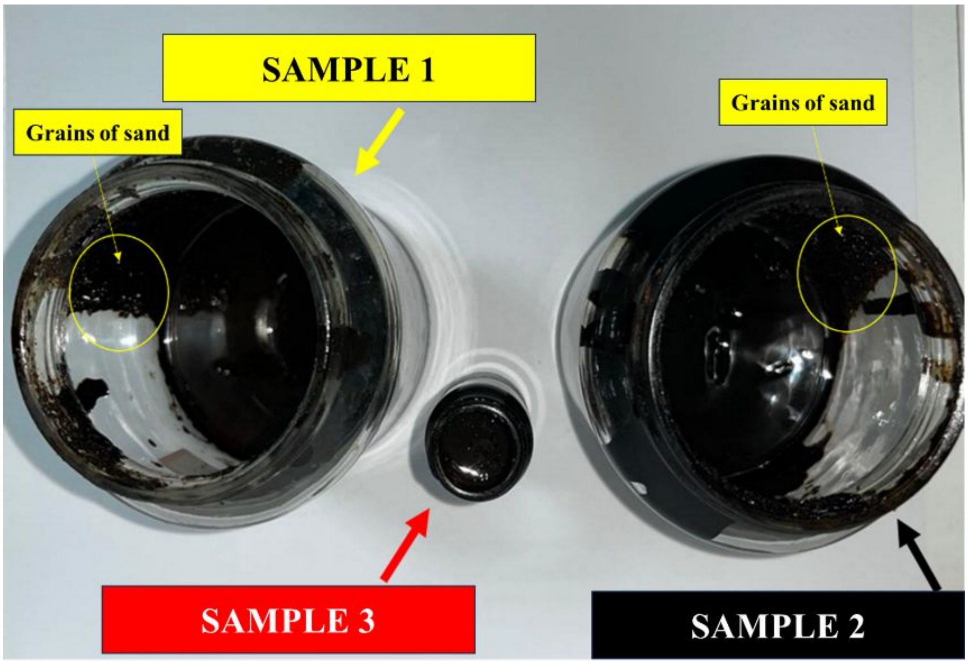

Figure S3. Chromatogram obtained by GC/MS/MS-MRM monitoring the transition of C27 steranes (m/z 372 > m/z 217).

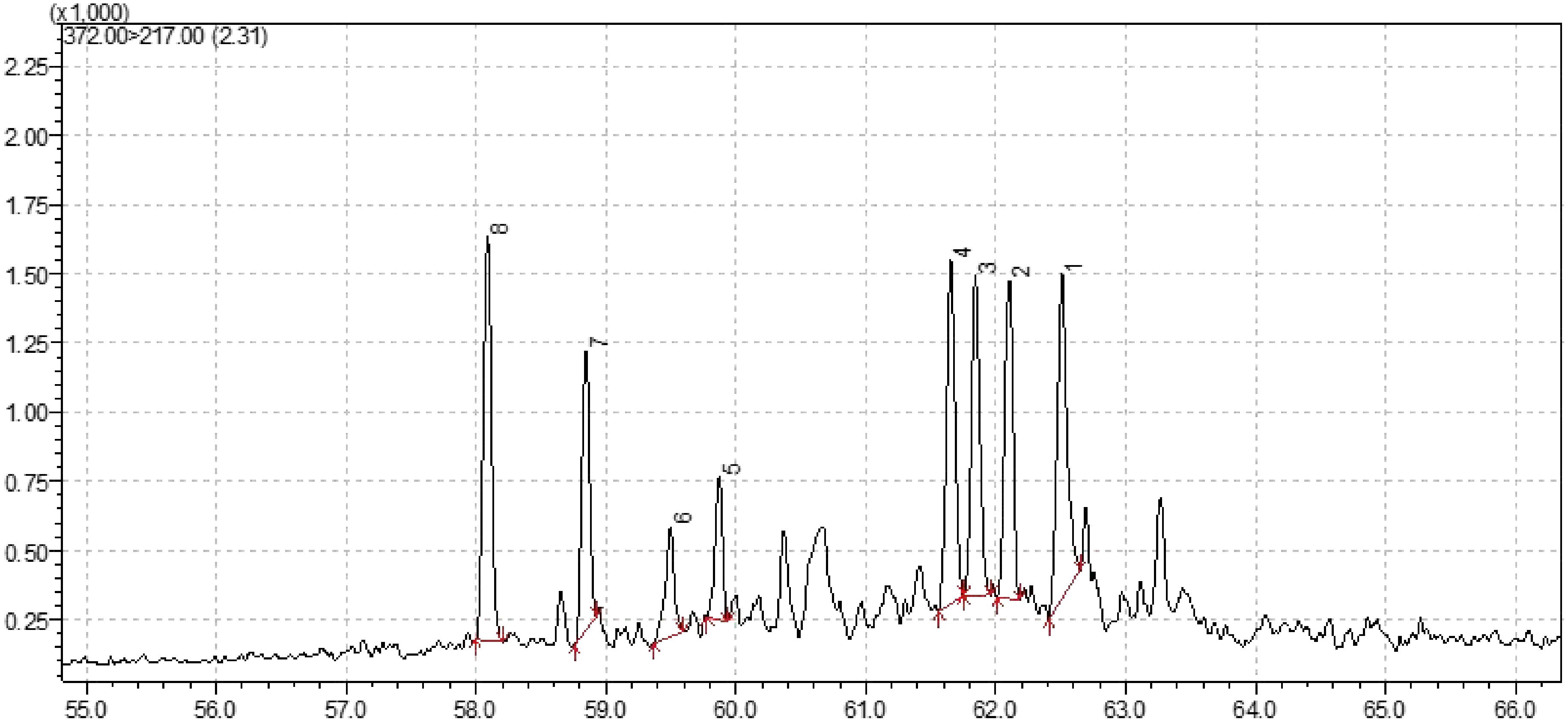

Figure S4. Chromatogram obtained by GC/MS/MS-MRM monitoring the transition of C28 steranes (*m/z* 386 > *m/z* 217).

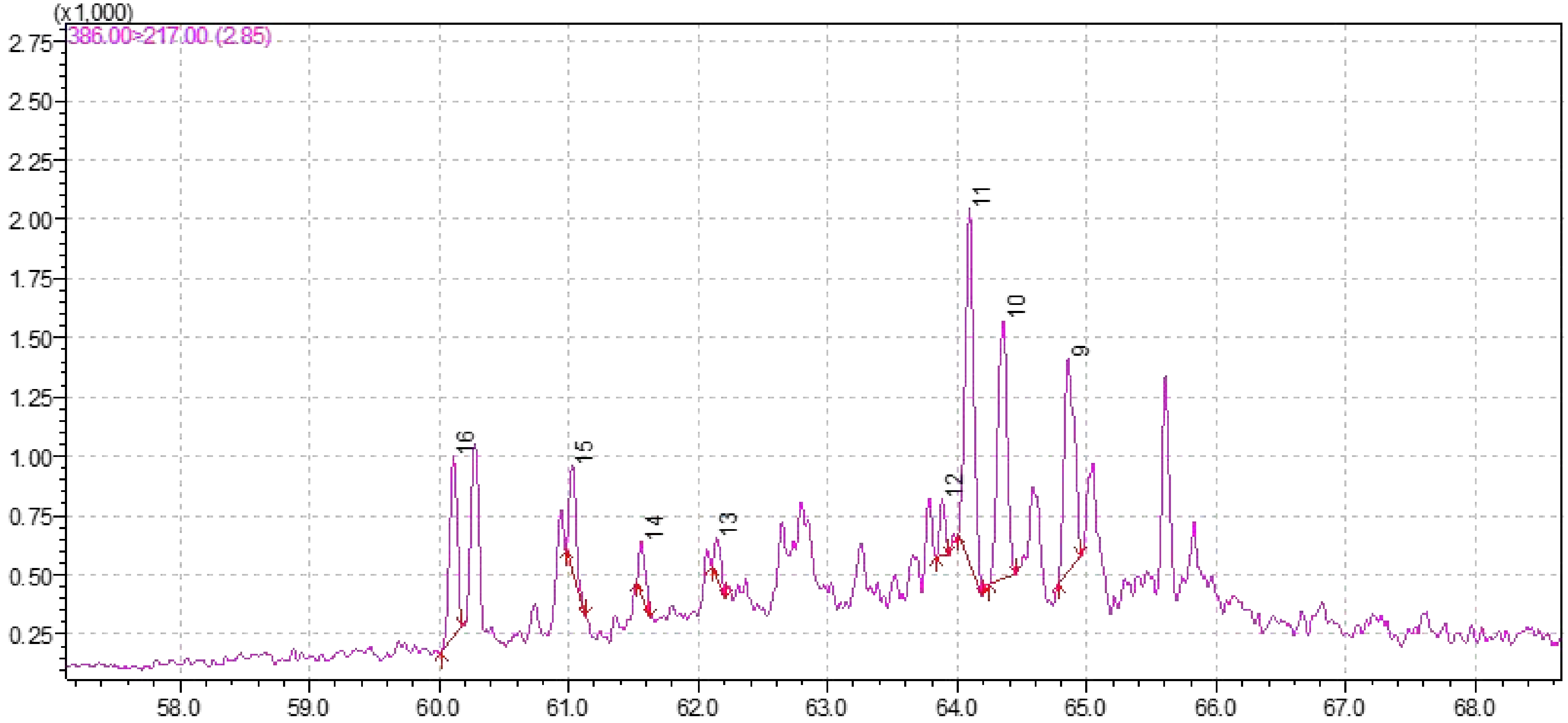

Figure S5. Chromatogram obtained by GC/MS/MS-MRM monitoring the transition of C29 steranes (*m/z* 400 > *m/z* 217).

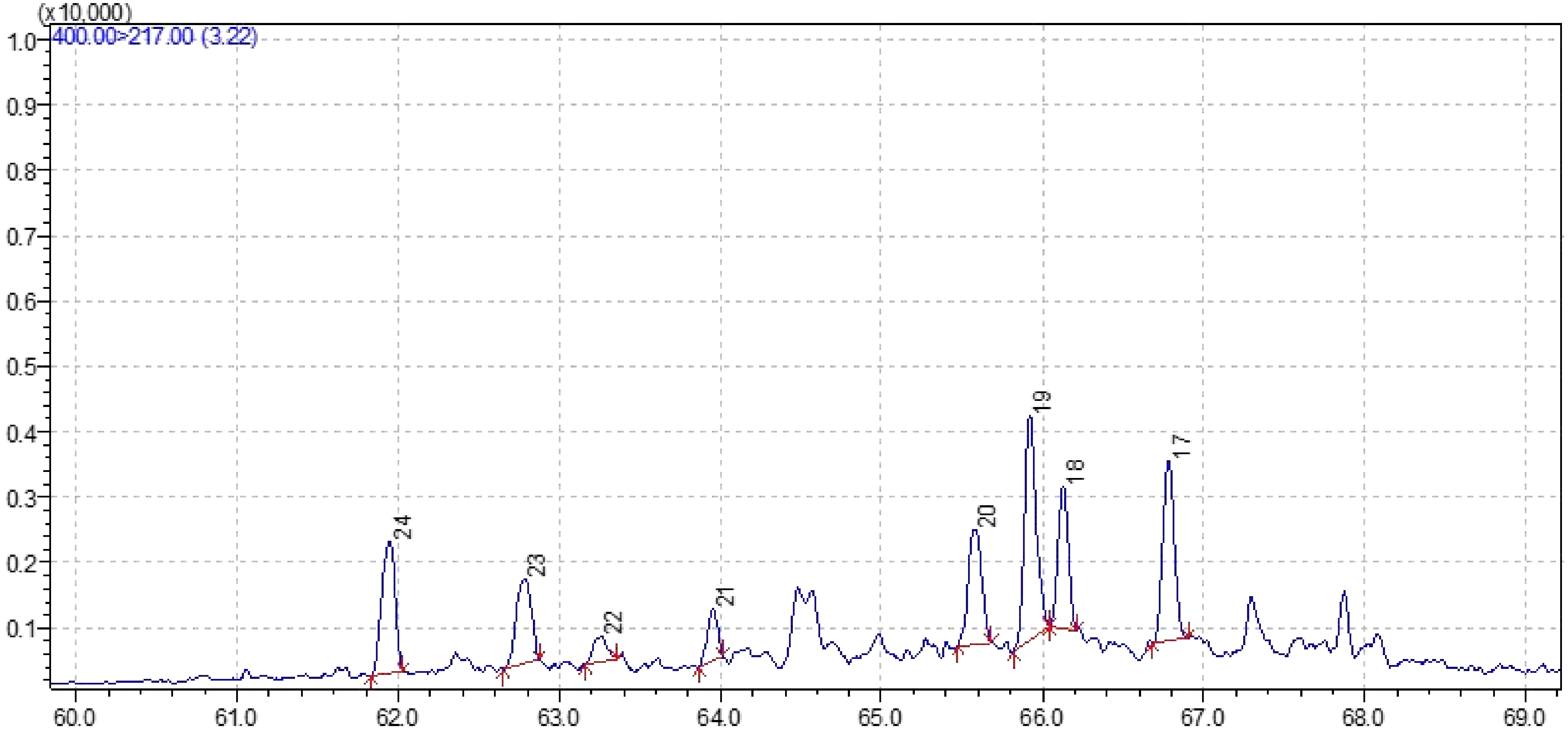

Figure S6. Chromatogram obtained by GC/MS/MS-MRM monitoring the transition of C30 steranes (*m/z* 414 > *m/z* 217).

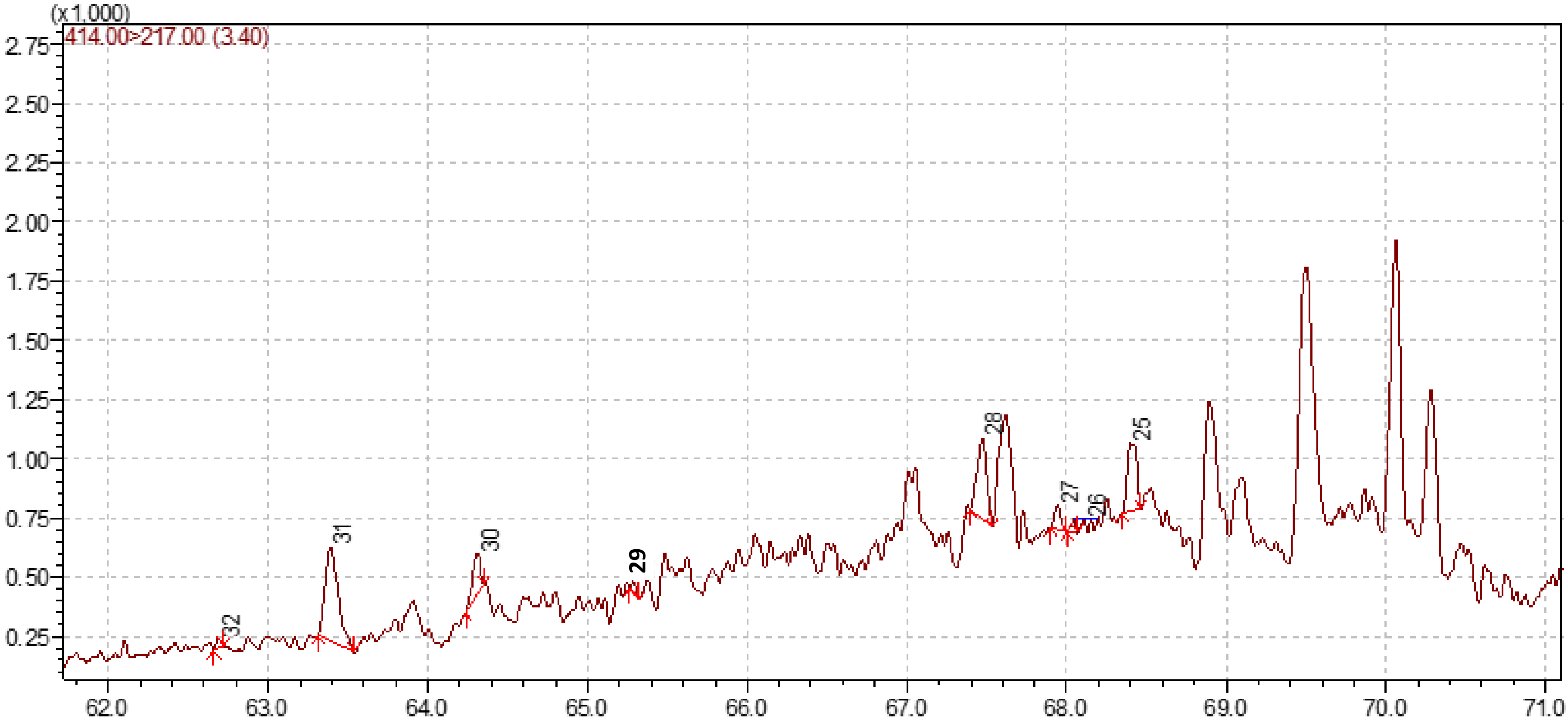

Figure S7. Chromatogram obtained by GC/MS/MS-MRM monitoring the transition of C27 diasteranes (*m/z* 414 > *m/z* 231).

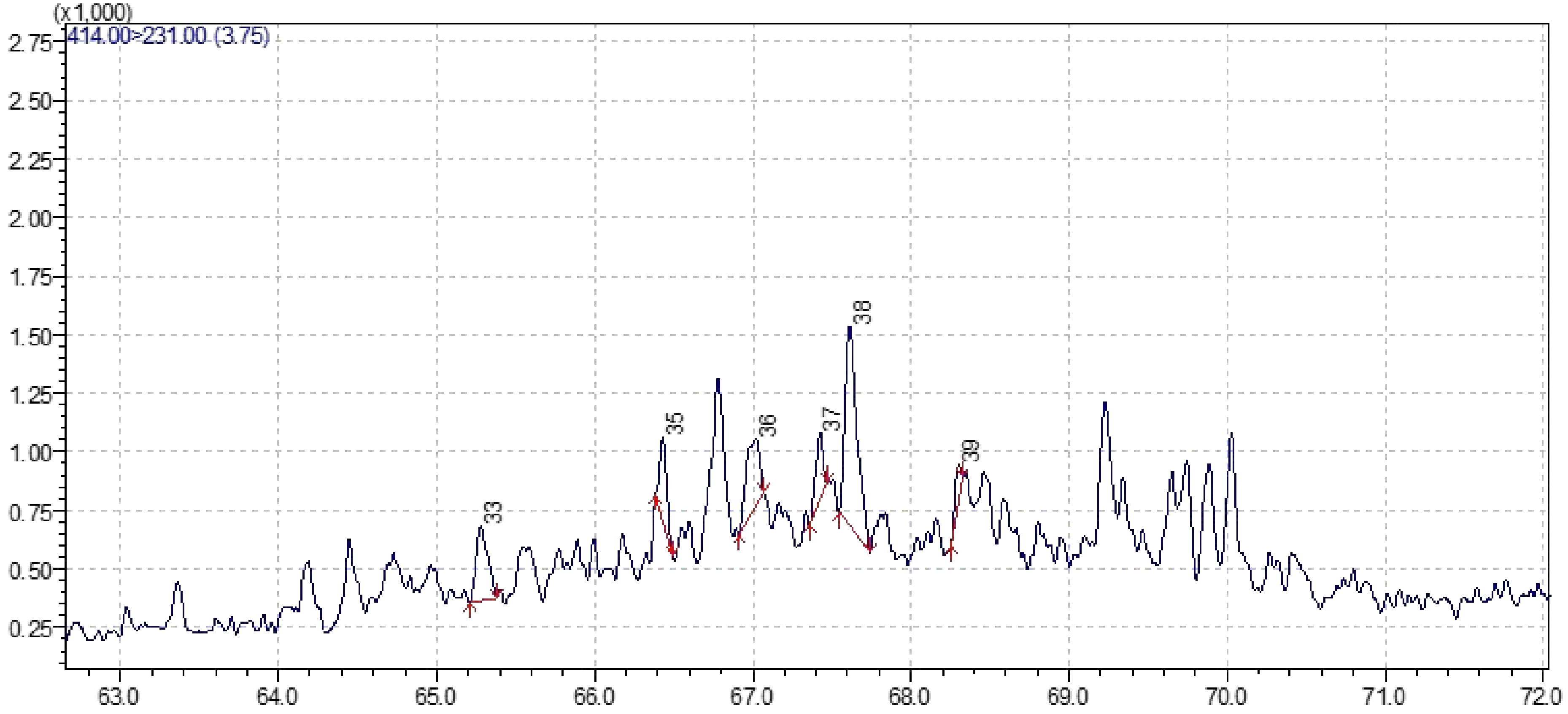

Figure S8. Chromatogram obtained by GC/MS/MS-MRM monitoring the transition of C27 hopanes (*m/z* 370 > *m/z* 191).

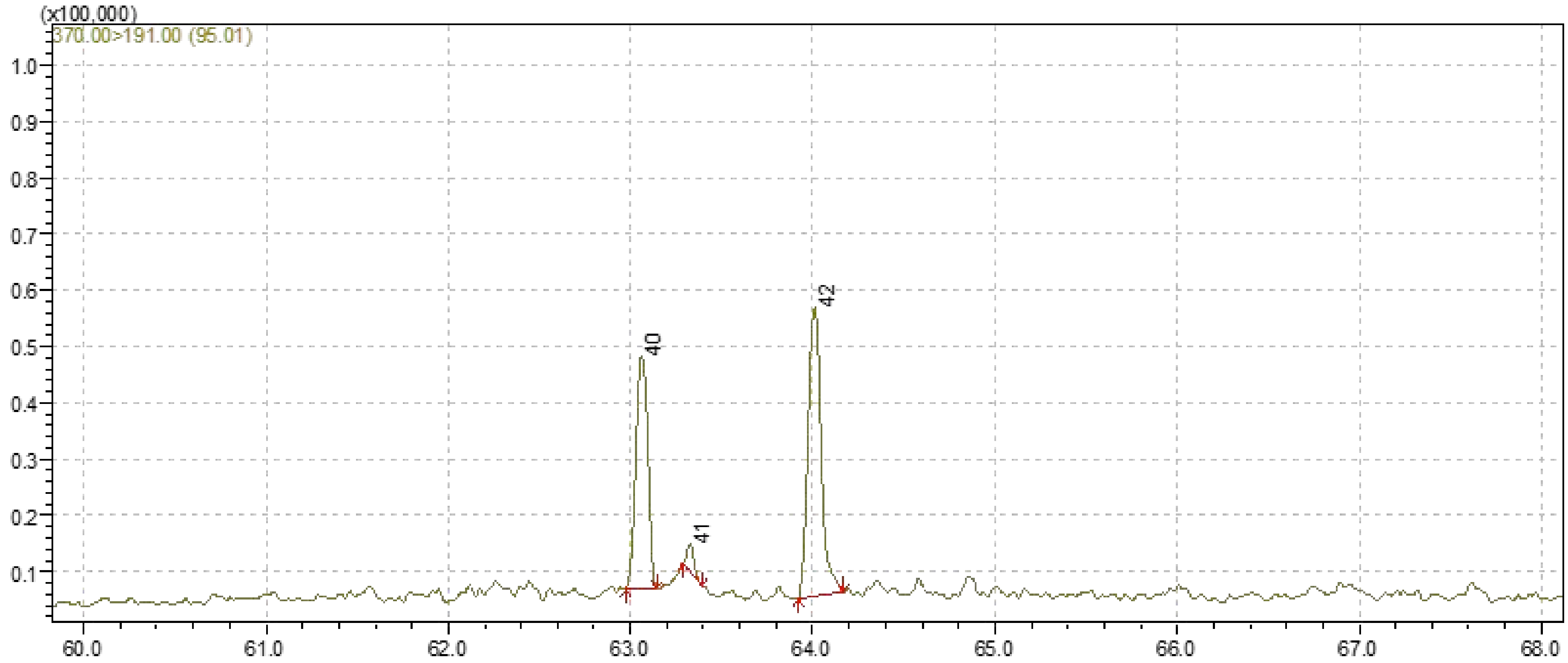

Figure S9. Chromatogram obtained by GC/MS/MS-MRM monitoring the transition of C29 hopanes (*m/z* 398 > *m/z* 191).

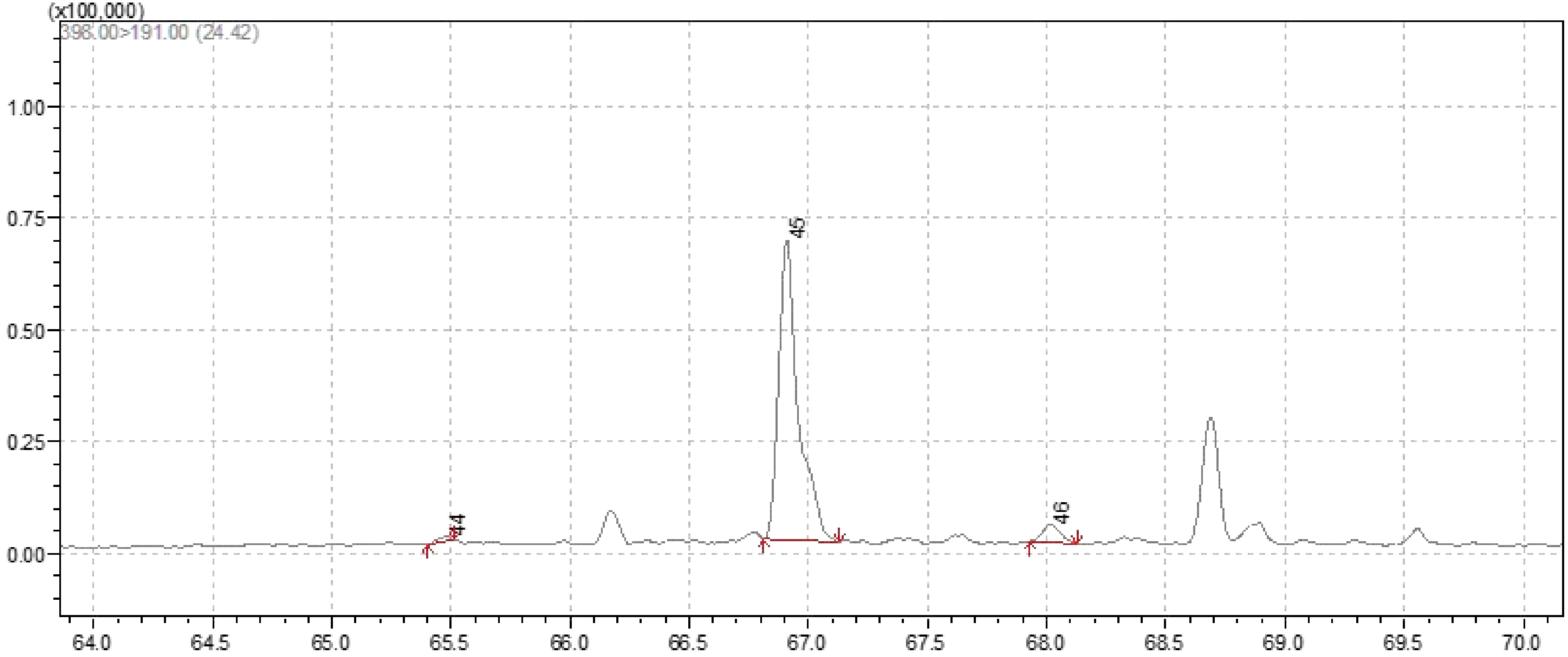

Figure S10. Chromatogram obtained by GC/MS/MS-MRM monitoring the transition of C30 hopanes (*m/z* 412 > *m/z* 191).

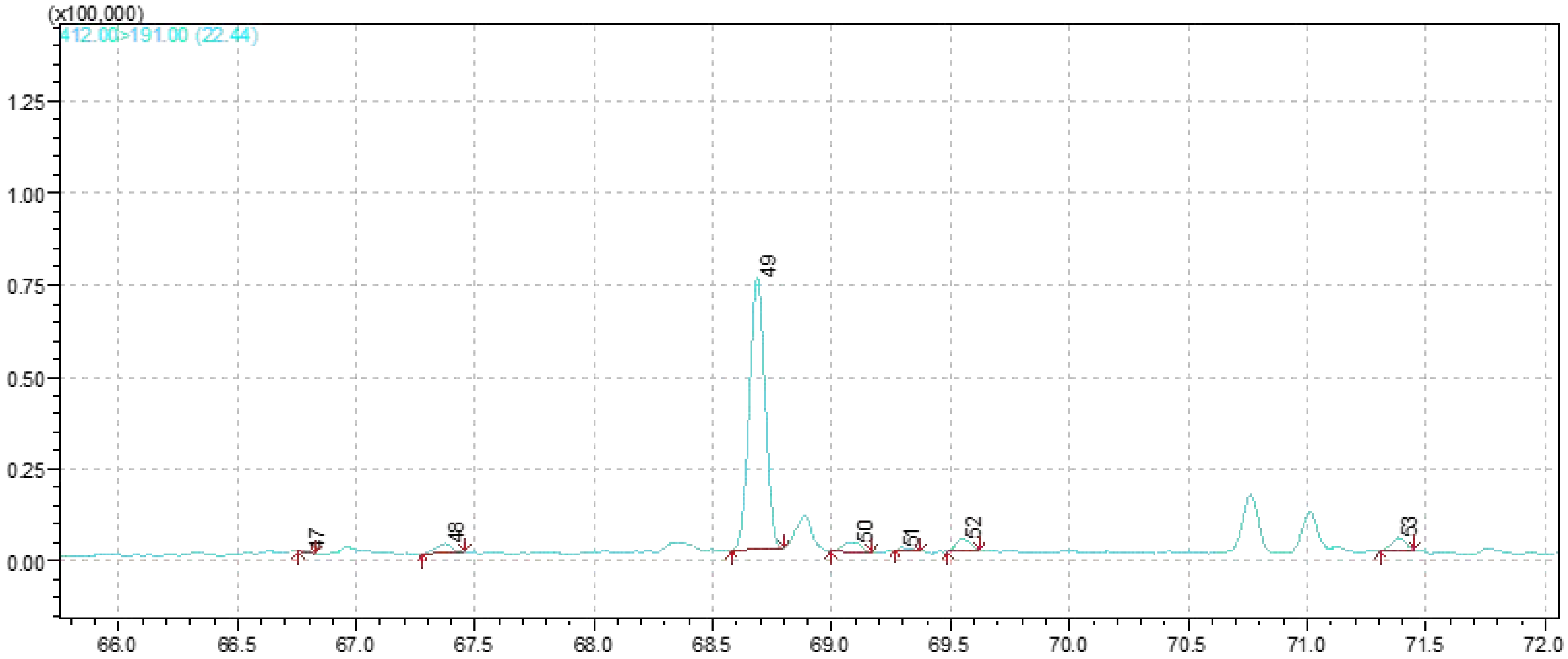

Figure S11. Chromatogram obtained by GC/MS/MS-MRM monitoring the transition of C31 hopanes (*m/z* 426 > *m/z* 191).

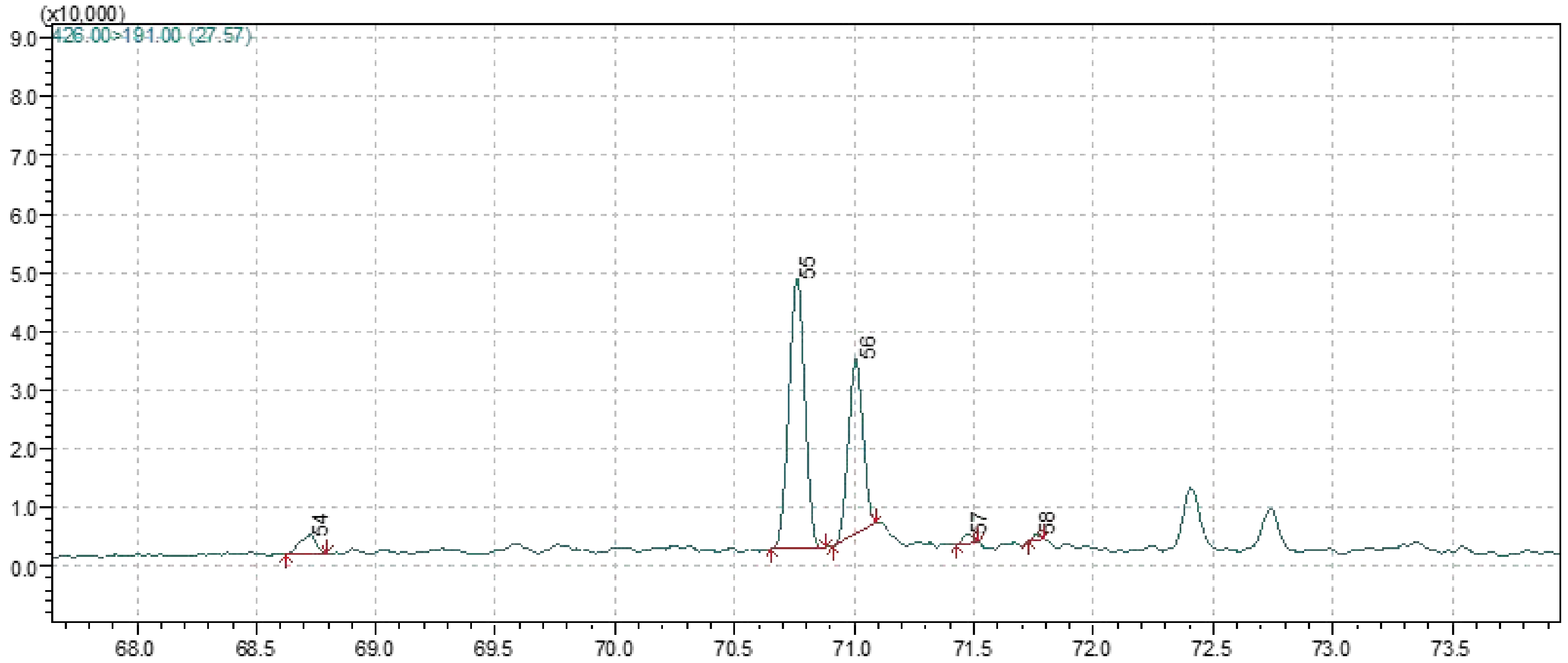

Figure S12. Chromatogram obtained by GC/MS/MS-MRM monitoring the transition of C32 hopanes (*m/z* 440 > *m/z* 191)

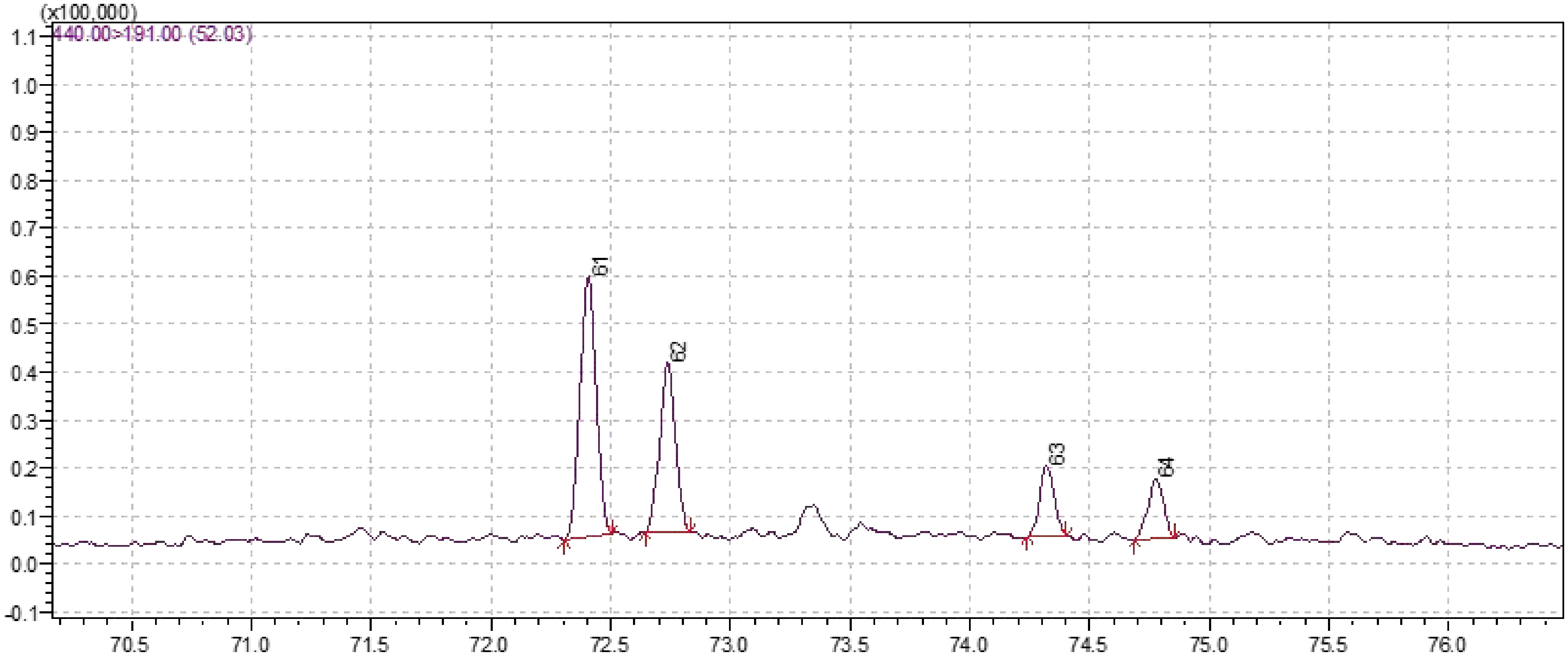

Table S1. Quality control of the data obtained by H-ESI(+)-FT-Orbitrap MS for the oil samples from the SE-AL basin, the drum and the spill

**Parameter**	**Sample A**	**Sample B**	**Sample C**	**Sample D**	**Sample T**	**Sample 1**	**Sample 2**	**Sample 3**
**Detected ions**	16772	13710	15443	15515	12010	14430	14647	14320
**Attributed ions**	8173	7483	7579	8047	7214	7983	8268	8072
**S/N > 3**	4000	3847	3836	4096	3013	2436	2467	2649
**Error < ±3 ppm**	477	327	287	400	354	352	287	340
**Error < ±2 ppm**	1487	1305	1490	1512	1178	910	921	998
**Error < ±1 ppm**	2036	2215	2059	2184	1481	1174	1259	1311

Figure S13. Histogram of groups obtained by H-ESI(+)-FT-Orbitrap MS for the oil spill samples.

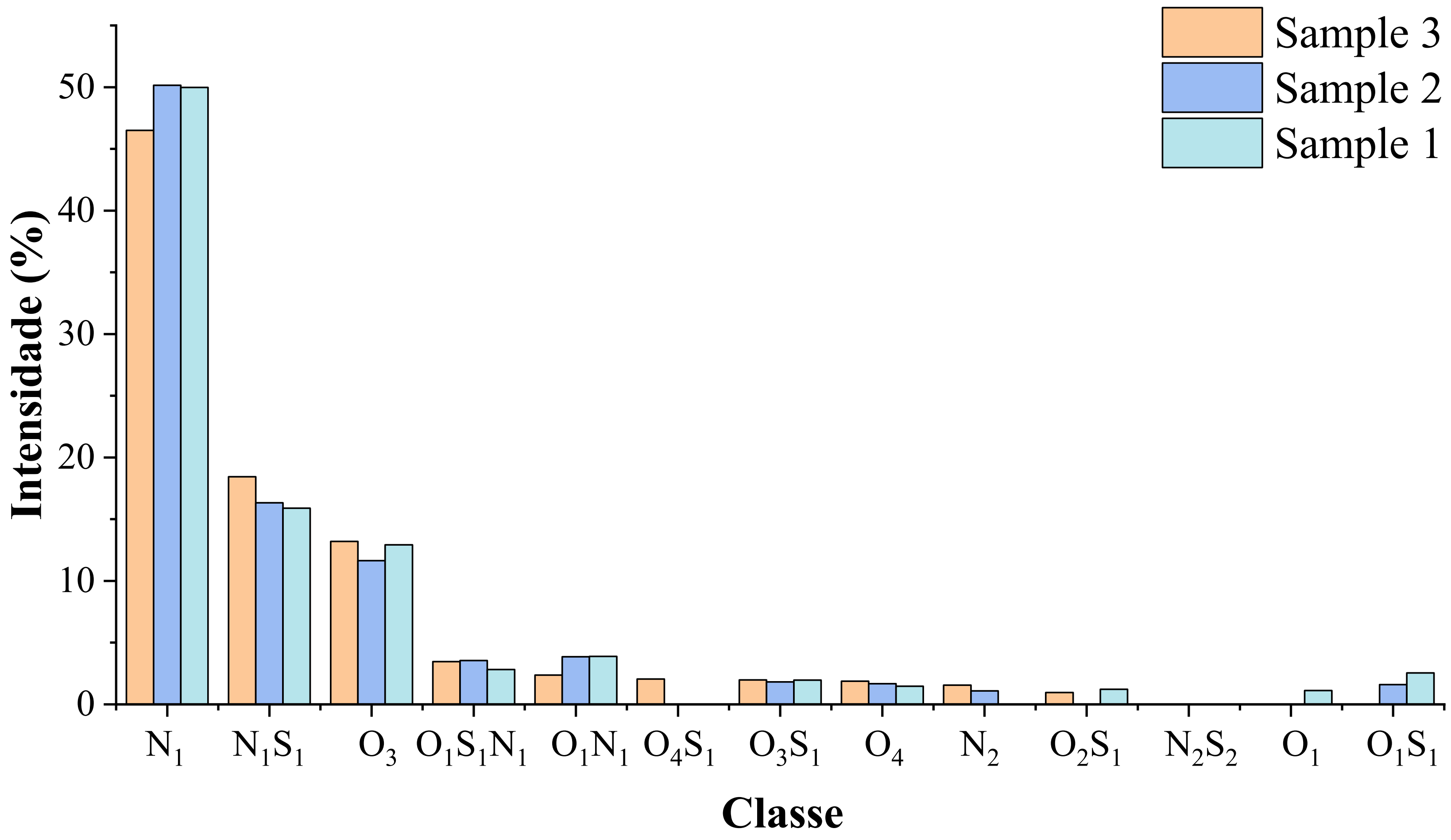

Supplement: Supplementary file 1 [file ao5c07056_si_001.pdf]
